# Supplementary material for: Oxidative stress‐induced phosphorylation of JIP4 regulates lysosomal positioning in coordination with TRPML1 and ALG2
Source: EMBO J. 2022 Oct 11;41(22):e111476. doi: 10.15252/embj.2022111476 (PMC9670204; doi:10.15252/embj.2022111476)
Supplement: Supplementary file 1 — Appendix [file EMBJ-41-e111476-s004.pdf]

Appendix

Oxidative stress-induced phosphorylation of JIP4 regulates lysosomal positioning in coordination with TRPML1 and ALG2

Yukiko Sasazawa, Sanae Souma, Norihiko Furuya, Yoshiki Miura, Saiko Kazuno, Soichiro Kakuta, Ayami Suzuki, Ryota Hashimoto, Hiroko Hirawake-Mogi, Yuki Date, Masaya Imoto, Takashi Ueno, Tetsushi Kataura, Viktor I. Korolchuk, Taiji Tsunemi, Nobutaka Hattori, Shinji Saiki

Table of contents

Appendix Figure S1. 2
Reaction scheme for enzymatic oxidation of spermine and spermidine.

Appendix Figure S2. 3
Spm and acrolein suppress mTORC1 signaling.

Appendix Figure S3. 4
Spm-induced lysosomal retrograde transport is mediated by both Ca^{2+} and PIKFyve.

Appendix Figure S4. 5
JIP4 is phosphorylated and translocated to lysosomes in response to spm treatment in SH-SY5Y cells.

Appendix Figure S5. 6
The purification of JIP4-myc-DDK and truncated His_6-JIP4 (100-300aa.)

Appendix Figure S6. 7
Characterization of JIP4 KO SH-SY5Y cells and the effect of ALG2 on lysosomal clustering.

Appendix Figure S7. 8
TMEM55B and TRPML1 mRNA levels are not altered in response to acrolein, H_2O_2 treatment and under starvation condition.

Appendix Table S1. 9
The amount of FDP-Lys in the sera of PD patients and healthy controls.

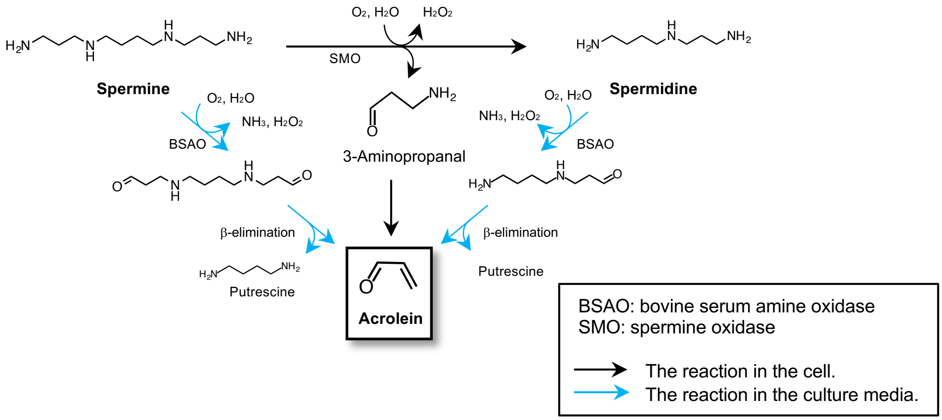

Appendix Figure S1. Reaction scheme for enzymatic oxidation of spermine and spermidine. In cell system, spm is metabolized to H_2O_2 and 3-aminopropanal by spermine oxidase (SMO), and then 3-aminopropanal spontaneously converted to acrolein. On the other hand, in the cultured media, bovine serum amine oxidase (BSAO) in the fetal bovine serum catalyzes the oxidative deamination of spermidine and spermine to produce an aminoaldehyde and an aminodialdehyde, respectively both of which spontaneously generate acrolein.

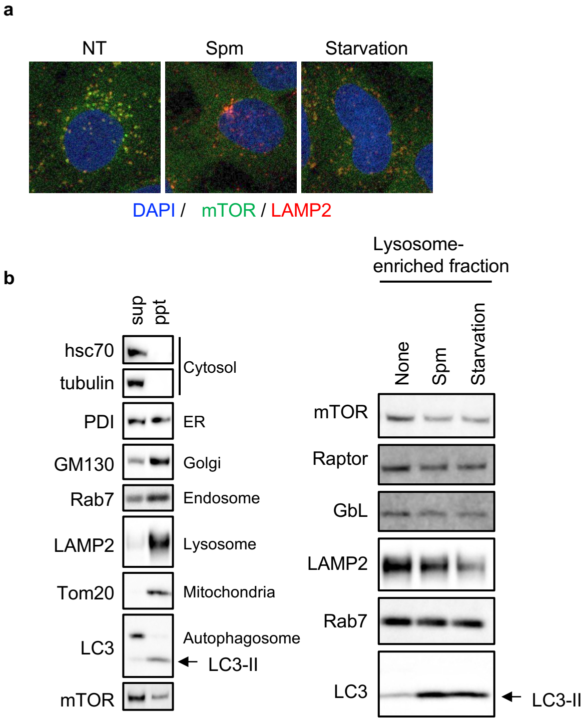

Appendix Figure S2. Spm and acrolein suppress mTORC1 signaling.
a. U2OS cells were treated with spm for 6 h or cultured in starvation medium for 1 h. Cells were fixed and stained with anti-LAMP2 (red) and anti-mTOR (green) antibodies. Nuclei were stained with DAPI (blue) **b.** SH-SY5Y cells were treated 50 µM spm or cultured in starvation medium for 1 h and then subjected to subcellular fractionation. Each fraction was immunoblotted with each organelle marker antibody (left). The supernatant (lysosome-enriched fraction) was immunoblotted with indicated antibodies (right).

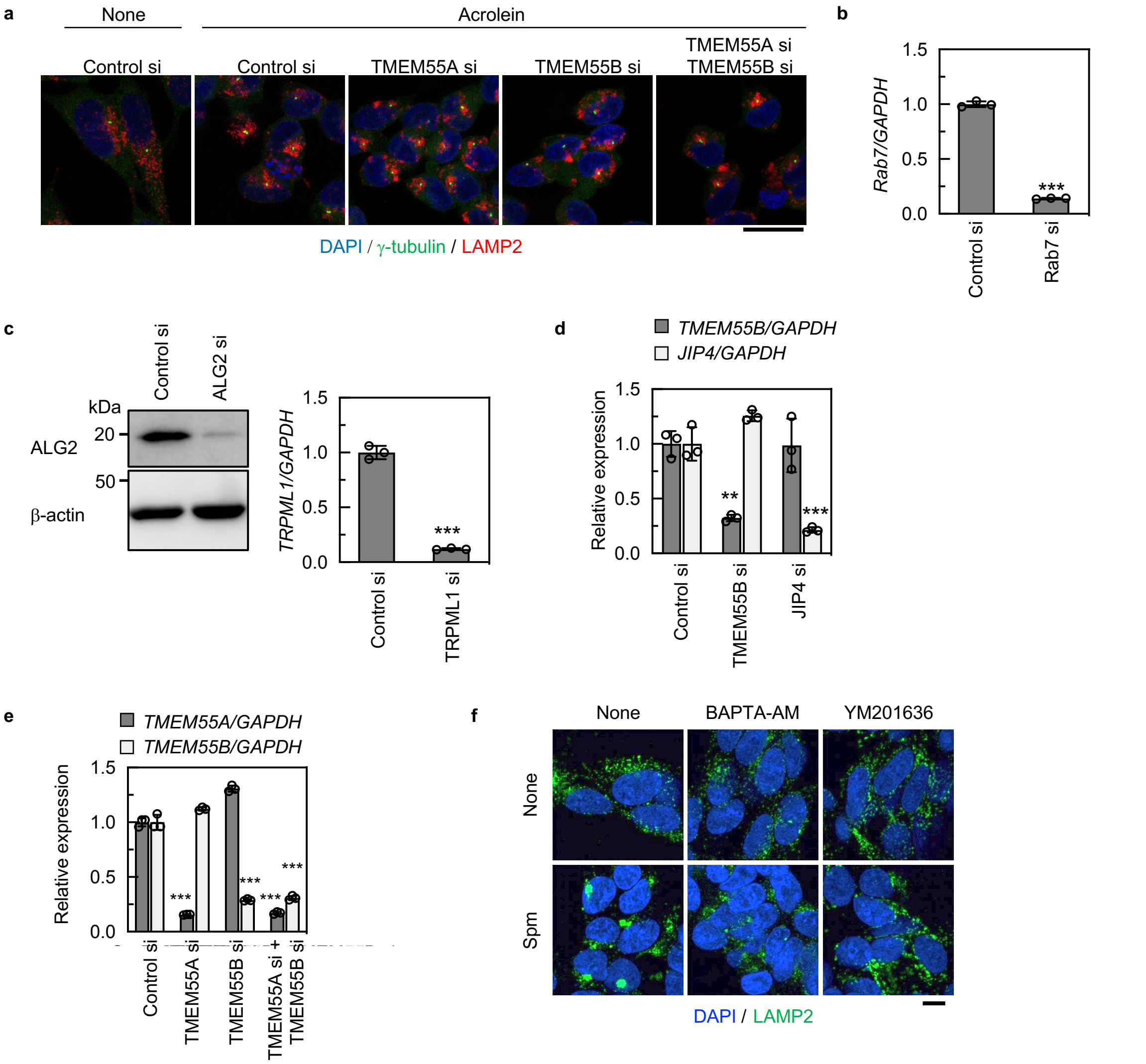

Appendix Figure S3. Spm-induced lysosomal retrograde transport is mediated by both Ca^{2+} and PIKFyve.

a. SH-SY5Y cells transfected with the indicated siRNAs for 48 h were treated with 50 μM acrolein for 2 h. Cells were fixed and stained with anti-LAMP2 (red) and anti-γ-tubulin (green) antibodies. Scale bar, 20 μm. **b–e.** SH-SY5Y cells were treated with the indicated siRNAs and knockdown efficiency of each siRNA was confirmed by qRT-PCR or western blotting. ***p<0.0001, **p<0.001 (b, c, Student t-test; d, e, Dunnett's test vs control si) n=3 technical replicates. **f.** SH-SY5Y cells were pretreated with 10 μM BAPTA-AM or 1 μM YM201636 for 1 h and then treated with 50 μM spm for an additional 4 h. Cells were fixed and stained with an anti-LAMP2 (green) antibody. Nuclei were stained with DAPI (blue). Scale bar, 10 μm.

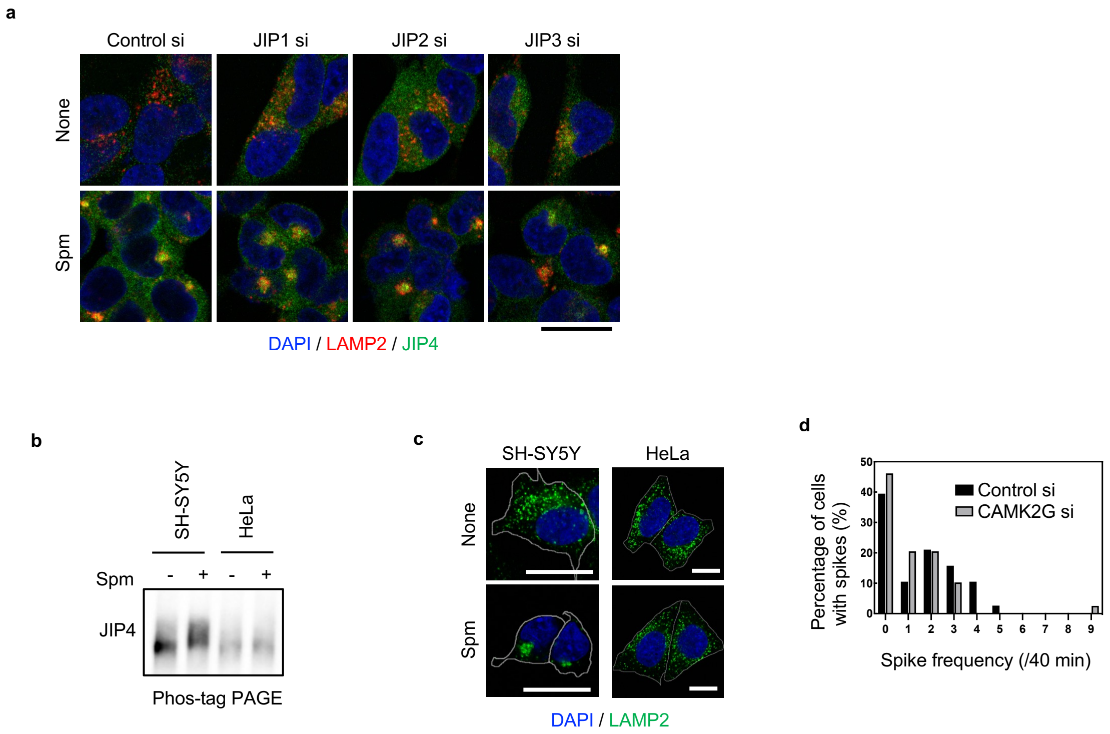

Appendix Figure S4. JIP4 is phosphorylated and translocated to lysosomes in response to spm treatment in SH-SY5Y cells.

a. SH-SY5Y cells transfected with the indicated siRNAs for 48 h were treated with 50 µM spm for 4 h. Cells were fixed and stained with anti-LAMP2 (red) and anti-JIP4 (green) antibodies. Scale bar, 20 µm. **b.** SH-SY5Y and HeLa cells were treated with 50 µM spm for 4 h. Cell lysates were subjected to Phos-tag PAGE and immunoblotted with an anti-JIP4 antibody. **c**. SH-SY5Y and HeLa cells were treated with 50 µM spm for 4 h. Cells were fixed and stained with an anti-LAMP-2 antibody (green). **d.** Cells transfected with control siRNA and CaMK2G siRNA for 48 h were loaded with Fura2-AM for 30 min. Cells were treated with 50 µM acrolein and observed by time-lapse fluorescence microscopy at 5-second intervals. Spikes with an amplitude of more than 0.05 for 40 min were counted. The value represents the percentage of cells with the indicated number of spikes to the total cells. Spike frequency "0" means no spikes in the cell.

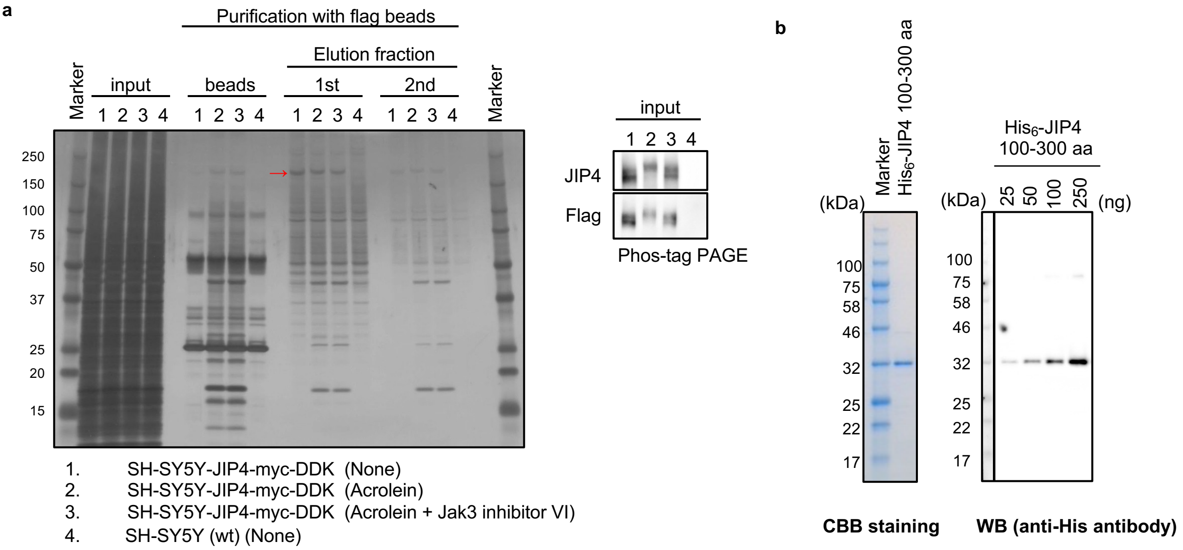

Appendix Figure S5. The purification of JIP4-myc-DDK and truncated His$_6$-JIP4 (100-300aa.)
a. JIP4-myc-DDK-expressing SH-SY5Y cells were treated with 40 µM acrolein with or without 10 µM Jak3 inhibitor VI for 2 h. Parental SH-SY5Y cells were used as a negative control. Cells were lysed and incubated with flag magnetic beads for 2 h. JIP4-myc-DDK protein was eluted by 1 mM flag peptide. The proteins in each fraction were visualized by Ag staining (left). The phosphorylation state was confirmed by Phos-tag PAGE and immunoblotting with anti-JIP4 and anti-flag antibodies (right). **b.** Purity check of purified recombinant his$_6$-tagged JIP4 (100-300 aa) by CBB staining and immunoblotting with anti-His tag antibody.

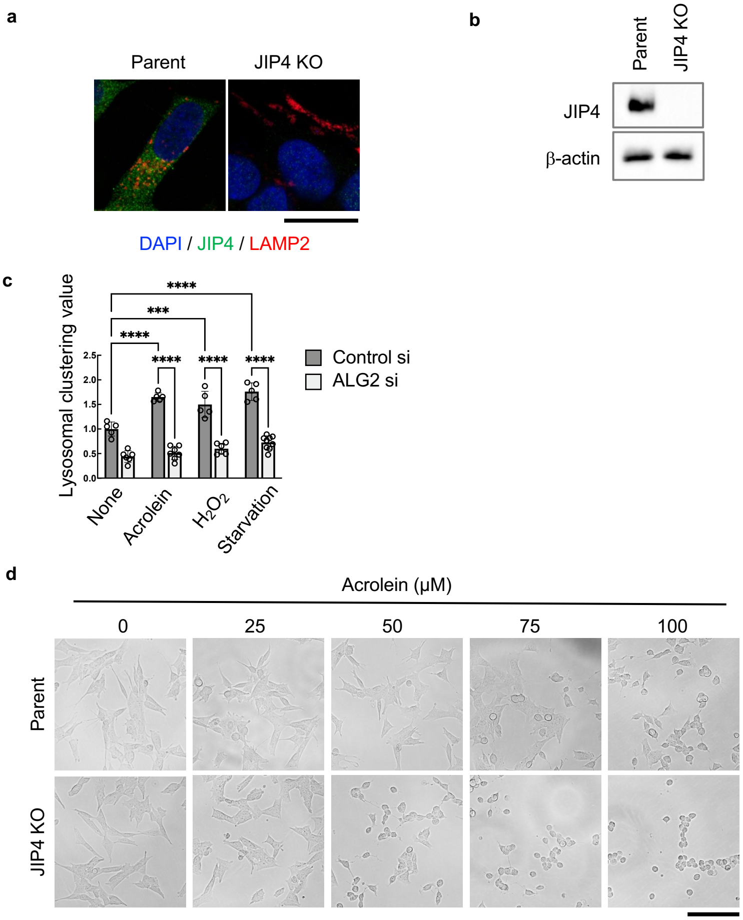

Appendix Figure S6. Characterization of JIP4 KO SH-SY5Y cells and the effect of ALG2 on lysosomal clustering.

a. Parental and JIP4 KO SH-SY5Y cells were fixed and stained with anti-LAMP2 (red) and anti-JIP4 (green) antibodies. Scale bar, 20 μm. **b.** Cell lysates from parental and JIP4 KO SH-SY5Y cells were immunoblotted with anti-JIP4 and anti-β-actin antibodies **c.** SH-SY5Y cells transfected with the ALG2 siRNAs for 48 h were treated with 50 μM acrolein, 500 μM H_2O_2 or under starvation for 2h. Cells were fixed and stained with anti-LAMP2 (red) and anti-γ-tubulin (green) antibodies. Clustered lysosomes were quantified as lysosomal clustering value. **** $p<0.0001$; *** $p<0.001$ N.S., not statistically different (Tukey-Kramer's test) 5-9 images were quantified per each condition. **d.** Parental and JIP4 KO SH-SY5Y cells were treated with various concentrations of acrolein for 6 h and then imaged by brightfield microscopy. Scale bar, 100 μm.

a

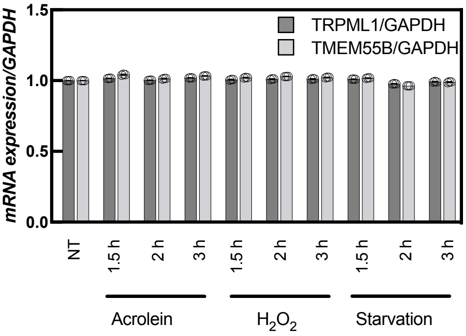

Appendix Figure S7. TMEM55B and TRPML1 mRNA levels are not altered in response to acrolein, H_2O_2 treatment and under starvation condition.

a. SH-SY5Y cells were treated with 50 μM acrolein, 500 μM H_2O_2 or starvation condition for indicated time. mRNA expression level of TMEM55B and TRPML1 were confirmed by qRT-PCR. n=3 technical replicates.

Appendix Table S1. The amount of FDP-Lys in the sera of PD patients and healthy controls.

	FDP-Lys [nmol/ml]	H&Y	age	gender (Male/Female)		FDP-Lys [nmol/ml]	H&Y	age	gender (Male/Female)
Ctrl 1	148.76	-	80	F	PD37	123.1	3	78	F
Ctrl 2	102.85	-	78	F	PD38	115.2	2	71	F
Ctrl 3	74.86	-	41	M	PD39	193.9	1	61	M
Ctrl 4	110.90	-	41	M	PD40	94.9	2	79	M
Ctrl 5	74.27	-	87	M	PD41	97.0	2	76	M
Ctrl 6	76.52	-	69	M	PD42	135.1	4	69	M
Ctrl 7	87.10	-	80	M	PD43	188.3	2	51	F
Ctrl 8	63.88	-	76	F	PD44	120.8	2	67	F
Ctrl 9	101.08	-	56	F	PD45	105.0	2	77	M
Ctrl 10	60.01	-	45	M	PD46	135.2	2	70	M
Ctrl 11	92.19	-	45	M	PD47	174.1	1	75	F
Ctrl 12	88.80	-	42	M	PD48	66.5	3	71	F
Ctrl 13	100.25	-	52	F	PD49	70.1	1	73	F
Ctrl 14	96.82	-	60	F	PD50	208.0	2	50	M
Ctrl 15	88.56	-	70	M	PD51	158.6	2	73	M
Ctrl 16	66.80	-	60	F	PD52	92.6	1	65	M
Ctrl 17	155.72	-	46	M	PD53	131.9	2	72	F
Ctrl 18	90.27	-	56	M	PD54	133.5	2	54	M
Ctrl 19	70.02	-	68	M	PD55	137.3	3	67	F
Ctrl 20	93.15	-	64	F	PD56	79.8	2	61	F
Ctrl 21	97.63	-	68	M	PD57	149.6	3	67	F
Ctrl 22	118.51	-	67	M	PD58	53.1	1	64	F
PD1	128.1	3	53	F	PD59	219.3	1	68	F
PD2	87.1	5	86	F	PD60	173.1	2	59	M
PD3	146.8	3	80	M	PD61	114.2	4	78	F
PD4	113.3	3	68	M	PD62	200.5	4	72	F
PD5	112.9	3	68	F	PD63	158.6	2	54	M
PD6	99.9	3	76	F	PD64	155.4	1	59	F
PD7	176.3	3	69	F	PD65	100.8	1	78	F
PD8	81.0	5	77	F	PD66	192.6	2	42	F
PD9	105.5	3	67	M	PD67	157.9	2	76	F
PD10	174.1	3	59	F	PD68	130.6	2	61	M
PD11	115.7	3	66	M	PD69	124.6	1	73	F
PD12	64.2	5	68	F	PD70	152.8	3	55	M
PD13	107.1	1	47	M	PD71	138.1	2	85	M
PD14	88.2	4	67	F	PD72	108.1	4	62	M
PD15	129.6	2	76	M	PD73	128.0	1	56	M
PD16	99.2	4	70	F	PD74	112.9	2	77	M
PD17	151.7	5	84	F	PD75	150.8	3	68	F
PD18	150.2	3	73	F	PD76	178.3	1	73	M
PD19	92.7	2	47	M	PD77	88.8	3	68	F
PD20	121.0	2	53	F	PD78	177.0	1	52	F
PD21	101.8	2	68	M	PD79	162.0	2	52	F
PD22	161.9	2	78	M	PD80	157.3	2	52	F
PD23	111.1	2	51	M	PD81	193.3	4	52	M
PD24	73.1	2	68	M	PD82	126.1	2	57	F
PD25	114.2	2	67	M	PD83	139.8	1	61	F
PD26	104.9	2	77	M	PD84	105.8	2	75	M
PD27	84.9	2	78	M	PD85	135.5	3	69	F
PD28	220.6	2	70	M	PD86	167.5	1	62	M
PD29	66.2	2	77	M	PD87	137.1	1	19	F
PD30	125.6	3	68	M	PD88	74.5	2	48	M
PD31	100.7	3	64	F	PD89	108.4	1	64	F
PD32	92.8	2	70	F	PD90	78.7	2	69	M
PD33	99.4	2	64	M	PD91	80.4	2	72	M
PD34	93.3	3	63	F	PD92	92.0	1	62	M
PD35	81.1	3	68	F	PD93	104.5	2	50	M
PD36	163.8	2	33	F	PD94	136.6	3	62	F
