# Supplementary material for: Oxidative stress‐induced phosphorylation of JIP4 regulates lysosomal positioning in coordination with TRPML1 and ALG2
Source: EMBO J. 2022 Oct 11;41(22):e111476. doi: 10.15252/embj.2022111476 (PMC9670204; doi:10.15252/embj.2022111476)
Supplement: Supplementary file 2 — Expanded View Figures PDF [file EMBJ-41-e111476-s003.pdf]

Expanded View Figures

Figure EV1. Spm/acrolein enhances autophagy and lysosomal retrograde transport.

A SH-SY5Y cells were treated with 40 μM acrolein and 100 nM bafilomycin A1 (BMA) for 2 h. Cell lysates were immunoblotted with the indicated antibodies.

B SH-SY5Y cells pre-treated with 1 mM aminoguanidine (AG) for 1 h were treated with 50 μM spm or spd for 4 h. Cell lysates were immunoblotted with the indicated antibodies.

C SH-SY5Y cells were treated with 50 μM spm for 4 h. Cells were fixed and stained with an anti-γ-tubulin (green) antibody to detect MTOC and an anti-LAMP2 (red) antibody. Nuclei were stained with DAPI (blue). Scale bar, 20 μm.

D, E SH-SY5Y cells were pre-treated with the indicated concentrations of nocodazole (D) or ciliobrevin D (E) for 1 h and then treated with 50 μM spm for an additional 4 h. Cells were fixed and stained with an anti-LAMP2 (green) antibody. Nuclei were stained with DAPI (blue). Scale bar, 20 μm.

F SH-SY5Y cells were treated with 50 μM spm or 100 nM bafilomycin A1 (BMA) for 4 h. Cathepsin B enzymatic activity was detected using a Magic Red-Cathepsin B assay kit. Scale bar, 20 μm.

G EGFP-galectin-3-expressing SH-SY5Y cells were treated with the indicated concentrations of acrolein and L-leucyl-L-leucine methyl ester (LLOMe; as a positive control). Lysosomal damage was detected by EGFP-galectin 3 accumulation. Scale bar, 20 μm.

Source data are available online for this figure.

©2022 The Authors

©2022 The Authors

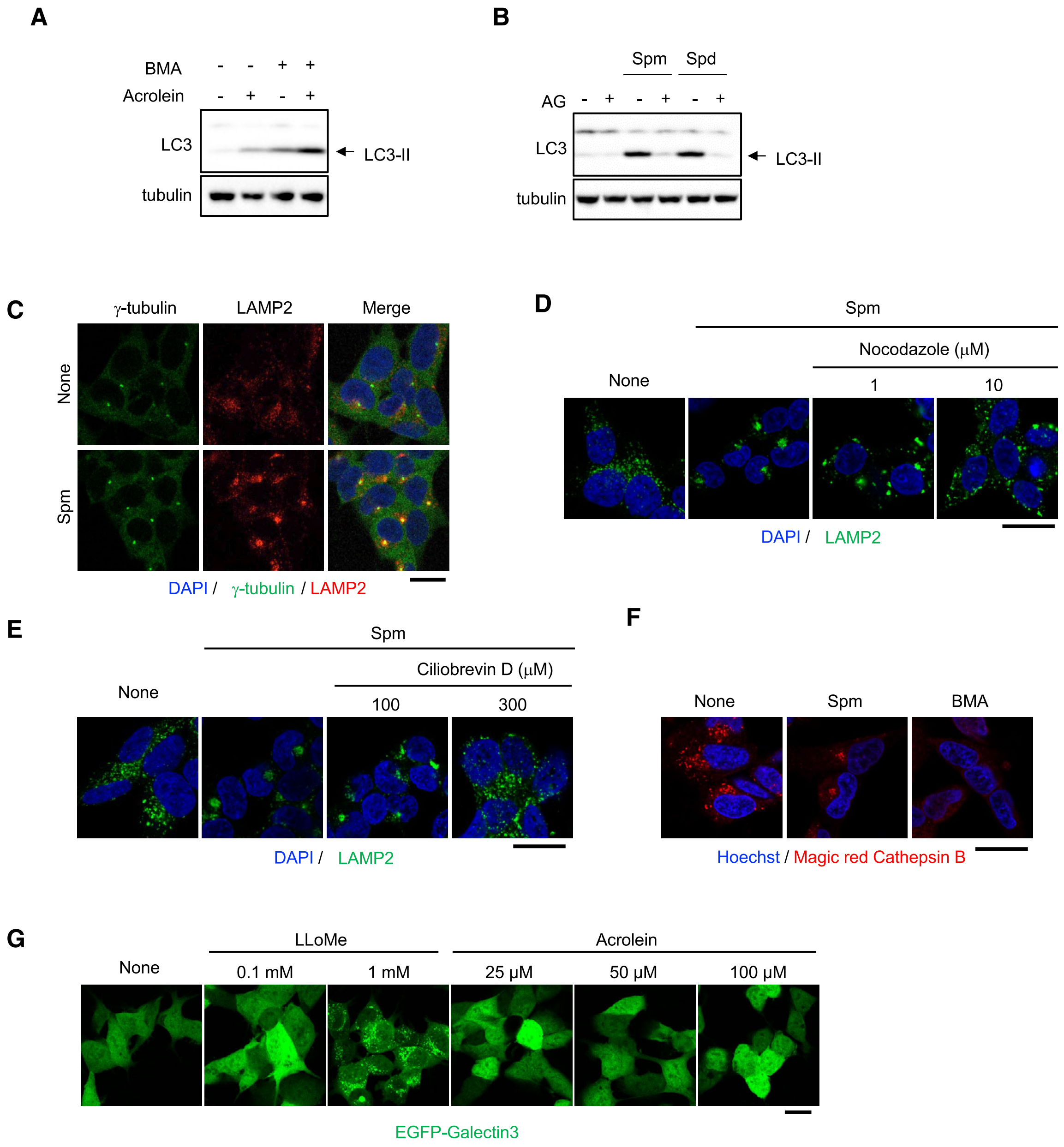
A
BMA - - + +
Acrolein - + - +
LC3
LC3-II
tubulin
B
Spm Spd
AG - + - + - +
LC3
LC3-II
tubulin
C
γ-tubulin LAMP2 Merge
None
Spm
DAPI / γ-tubulin / LAMP2
D
Spm
Nocodazole (μM)
None 1 10
DAPI / LAMP2
E
Spm
Ciliobrevin D (μM)
None 100 300
DAPI / LAMP2
F
None Spm BMA
Hoechst / Magic red Cathepsin B
G
LLoMe Acrolein
None 0.1 mM 1 mM 25 μM 50 μM 100 μM
EGFP-Galectin3

Figure EV1.

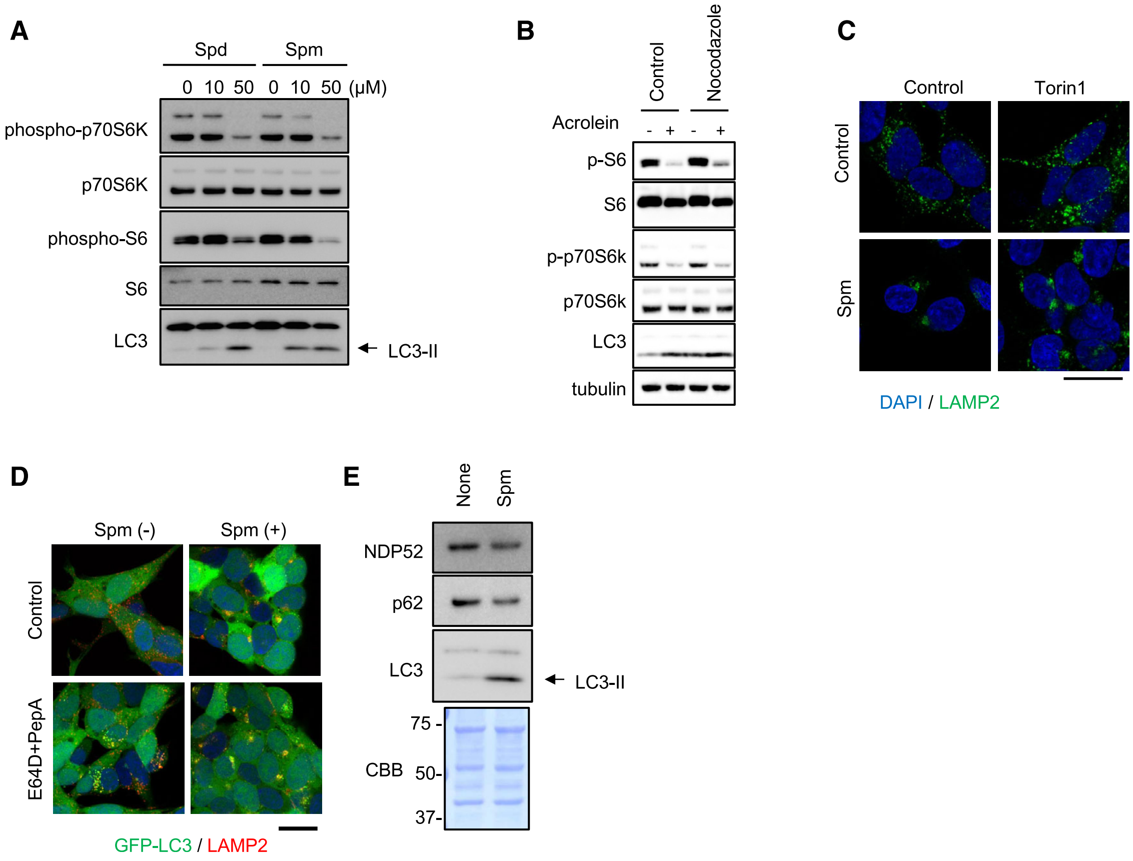

Figure EV2. Spm/acrolein suppresses mTORC1 signalling.

A SH-SY5Y cells were treated with spd or spm for 4 h. Cell lysates were immunoblotted with the indicated antibodies.
B SH-SY5Y pre-treated with 10 μM nocodazole for 1 h were then treated with 50 μM acrolein for 2 h. Cell lysates were immunoblotted with the indicated antibodies.
C SH-SY5Y were treated with 200 nM Torin1 with or without 50 μM spm for 4 h. Cells were fixed and stained with an anti-LAMP2 (green) antibody. Nuclei were stained with DAPI (blue). Scale bar, 20 μm.
D SH-SY5Y cells stably expressing GFP-LC3 were pre-treated with 10 μg/ml E64D plus pepstatin A for 2 h and then treated with 50 μM spm for an additional 4 h. Cells were fixed and stained with an anti-LAMP2 (red) antibody. Nuclei were stained with DAPI (blue). Scale bar, 20 μm.
E SH-SY5Y cells were treated with spm for 4 h. Cell lysates were immunoblotted with the indicated antibodies. CBB staining was used as a loading control.

Source data are available online for this figure.

 ©2022 The Authors

Figure EV3. Spm/acrolein-induced lysosomal clustering and JIP4 phosphorylation are mediated by CaMK2G.

- A Cells were transfected with the TMEM55B-myc-DDK plasmid for 24 h and then treated with 50 μM spm for 4 h. Cell lysates were immunoprecipitated with flag magnetic beads and immunoblotted with the indicated antibodies.
- B SH-SY5Y cells were treated with 50 μM spm for 4 h. Cells were fixed and stained with anti-JIP4 (green) and anti-LAMP2 (red) antibodies. Nuclei were stained with DAPI (blue). Scale bar, 20 μm.
- C SH-SY5Y cells were pre-treated with 0.1 mM N-acetyl-L-cysteine (NAC) for 1 h and then treated with 50 μM acrolein for an additional 2 h. Cells were fixed and stained with anti-LAMP2 (green) and anti-JIP4 antibodies. Nuclei were stained with DAPI (blue). Scale bar, 20 μm.
- D SH-SY5Y cells treated with 50 μM spm for 4 h were lysed, subjected to Phos-tag PAGE and immunoblotted with an anti-JIP4 antibody.
- E SH-SY5Y cells treated with 50 μM spm were lysed and treated with lambda phosphatase (PPase) for 60 min. The reactant was subjected to Phos-tag PAGE and immunoblotted with an anti-JIP4 antibody.
- F, G SH-SY5Y cells pre-treated with 10 μM JAK3 inhibitor VI (E) and 5 μM Gö6976 (F) for 1 h were treated with 50 μM spm for an additional 4 h. Cells were fixed and stained with anti-JIP4 (green) and anti-LAMP2 antibodies (red) (scale bar, 20 μm).
- H SH-SY5Y cells were pre-treated with 10 μM Jak3 inhibitor VI for 1 h and then treated with 50 μM spm for an additional 4 h. Cells lysates were subjected to Phos-tag PAGE and immunoblotted with an anti-JIP4 antibody.
- I SH-SY5Y cells transfected with Jak3 siRNA for 48 h were treated with 50 μM spm for 4 h. Cells were fixed and stained with an anti-LAMP2 antibody (red). Scale bar, 20 μm.
- J SH-SY5Y cells pre-treated with 1 μM Gö7874 for 1 h were treated with 50 μM spm for an additional 4 h. Cells were fixed and stained with anti-JIP4 (green) and anti-LAMP2 antibodies (red) (scale bar, 20 μm).
- K, L SH-SY5Y cells transfected with the indicated siRNAs for 48 h were treated with 50 μM spm for 4 h. (K) Cell lysates were subjected to Phos-tag PAGE and immunoblotted with an anti-JIP4 antibody. (L) Cells were fixed and stained with anti-LAMP2 (red) and anti-JIP4 (green) antibodies. Scale bar, 20 μm.

Source data are available online for this figure.

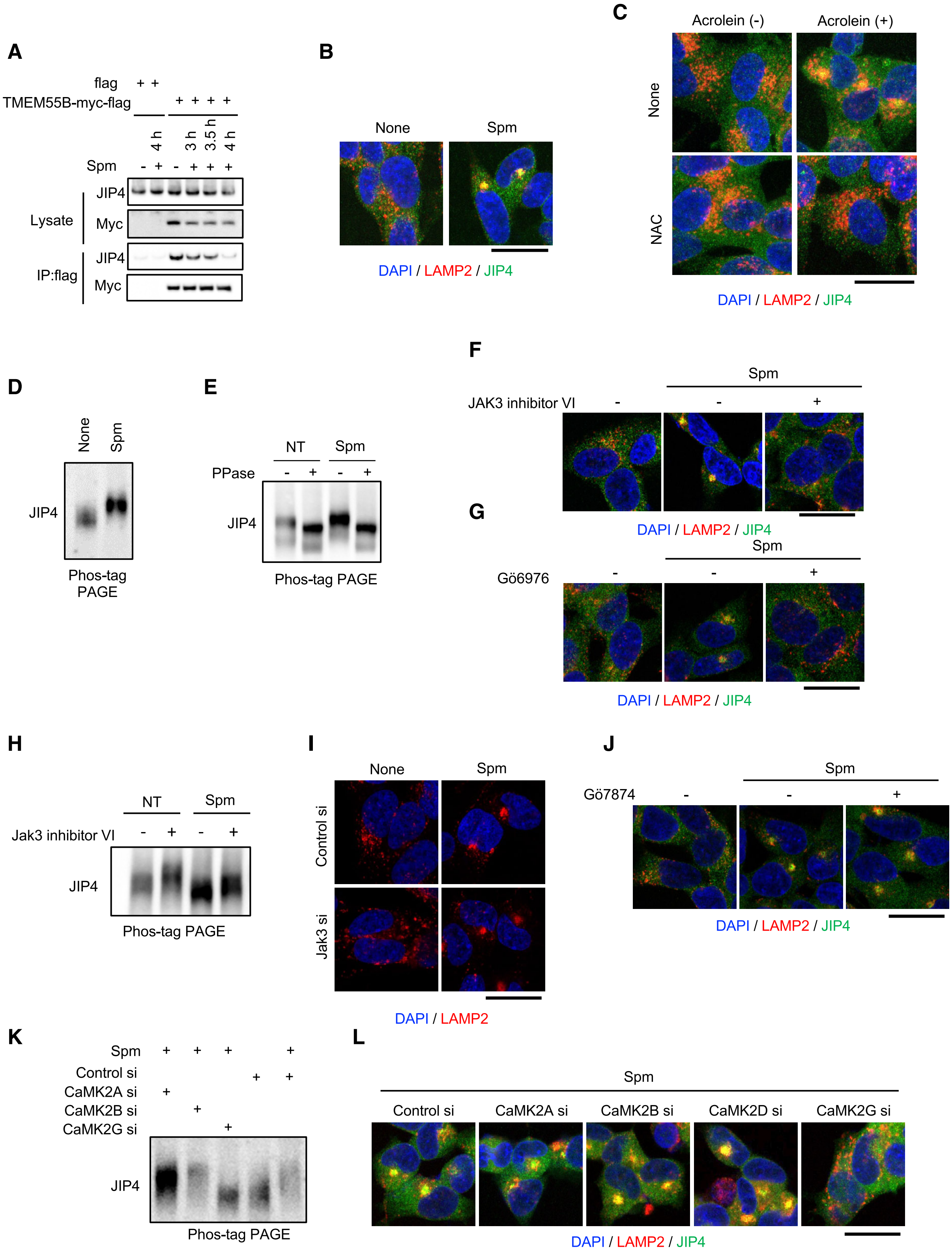

Figure EV3.

 ©2022 The Authors

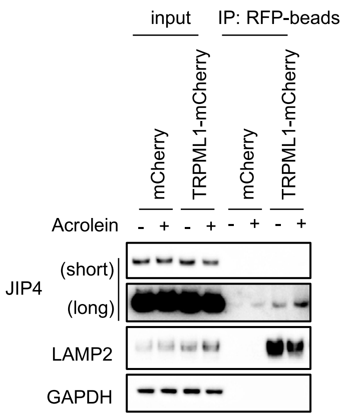

Figure EV4. JIP4 translocates to the lysosome in response to acrolein treatment.

mCherry and TRPML1-mCherry stably expressing SH-SY5Y cells were treated with 50 µM acrolein for 2 h. Lysosomes were isolated by Lyso-IP using anti-RFP magnetic beads. Each fraction was immunoblotted with indicated antibodies.

Source data are available online for this figure.

A

```
    JIP4_HUMAN   ERPISLGIFPLPAGDGLLTPDAQKG-GETPGSEQWKFQ
    JIP4_MOUSE   ERPISLGIFPLPAGDGLLTPDTQKG-GETPGSEQWKFQ
     SPAG9_RAT   ERPISLGIFPLPAGDGLLTPDTQKG-GETPGSEQWKFQ
   SPAG9_BOVIN   ERPISLGIFPLPSGDGLLTPDTQKG-GETPGSEQWKFQ
 SPAG9_CHICKEN   ERPISLGIFPLPPGDALLTPEAQREAGETPGSEHWKFH
  SPAG9a_DANRE   ERPLSLGIFPMSAGGSSLTPDVQGR-AETPGMEGWRFN
                 *** ******   *   ***  *    **** *  * *

    JIP1_HUMAN   ----------------------------------PA
    JIP2_HUMAN   LRLTTLGAQDSLNNNGGFDLVRPASWQETALCSPAPE
    JIP3_HUMAN   ERPTSLNVFPLADGT---VRAQIGGKLVPAGDHWHLS
    JIP4_HUMAN   ERPISLGIFPLPAGDGLLTPDAQKGGETPGSEQWKFQ
                  *   *
```

B

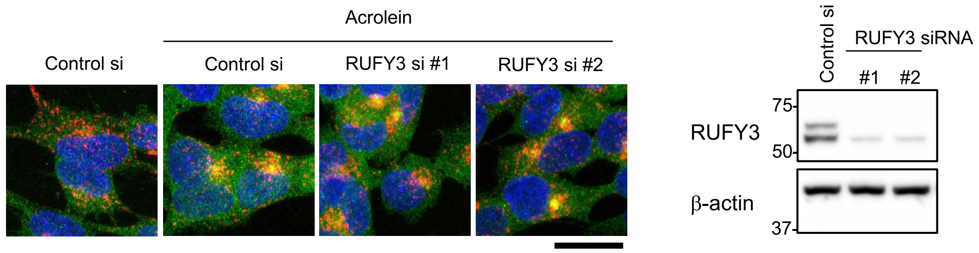

Figure EV5. RUFY3 is not involved in acrolein-induced lysosomal clustering.

A The sequence alignment analyses of JIP4 or JIP4 homologues among vertebrate species (upper), and human JIP1, JIP2, JIP3 and JIP4 (lower). Common amino acids were indicated by asterisks. T217 in human JIP4 and its homologous amino acids were underlined.

B SH-SY5Y cells transfected with the indicated siRNAs for 48 h were treated with 50 µM acrolein for 2 h. (left) Cells were fixed and stained with anti-LAMP2 (red) and anti-JIP4 (green) antibodies. Scale bar, 20 µm. (Right) The knockdown efficiency of each siRNA was assessed by immunoblotting with anti-RUFY3 antibody.

Source data are available online for this figure.

©2022 The Authors
