# Supplementary material for: Oxidative stress‐induced phosphorylation of JIP4 regulates lysosomal positioning in coordination with TRPML1 and ALG2
Source: EMBO J. 2022 Oct 11;41(22):e111476. doi: 10.15252/embj.2022111476 (PMC9670204; doi:10.15252/embj.2022111476)
Supplement: Supplementary file 4 — Movie EV2 [file EMBJ-41-e111476-s012.zip › Movie EV2/Movie EV 2 legend.docx]

**Movie EV2**

Live cell calcium imaging of SH-SY5Y cells transfected with the control siRNA during acrolein treatment.
