# Supplementary material for: Oxidative stress‐induced phosphorylation of JIP4 regulates lysosomal positioning in coordination with TRPML1 and ALG2
Source: EMBO J. 2022 Oct 11;41(22):e111476. doi: 10.15252/embj.2022111476 (PMC9670204; doi:10.15252/embj.2022111476)
Supplement: Supplementary file 6 — Source Data for Expanded View and Appendix [file EMBJ-41-e111476-s014.zip › Appendix figures/gel image_Appendix Figs.pdf]

Source data for Appendix Fig. S2

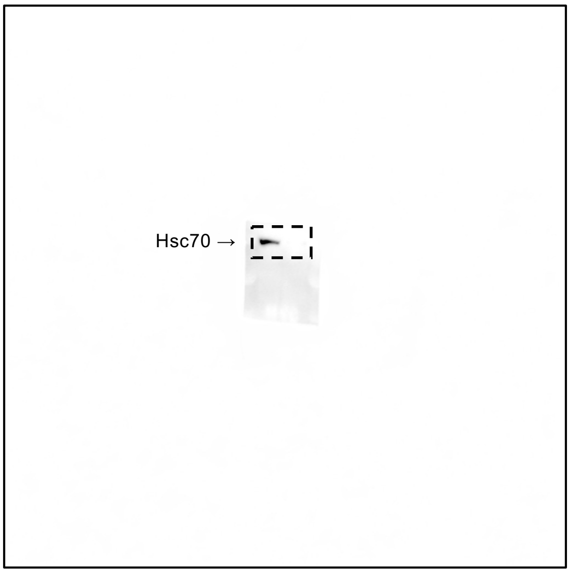

Full unedited image for Appendix Fig. S2b (left), Hsc70.

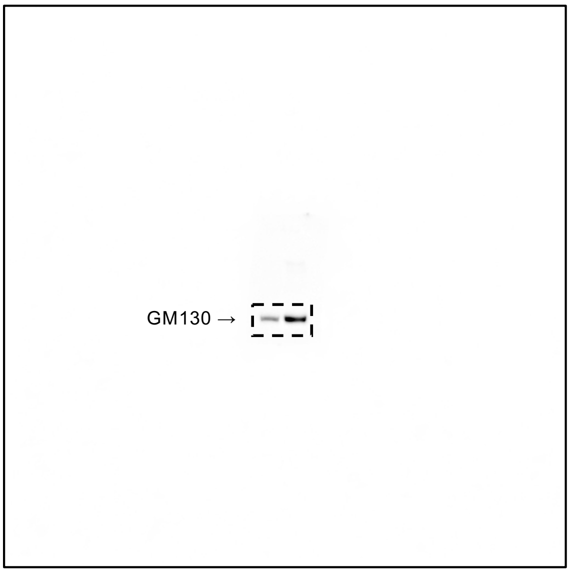

Full unedited image for Appendix Fig. S2b (left), GM130.

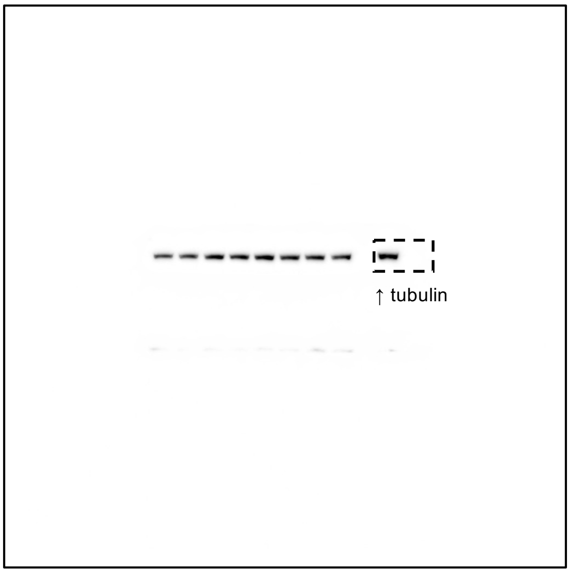

Full unedited image for Appendix Fig. S2b (left), tubulin.

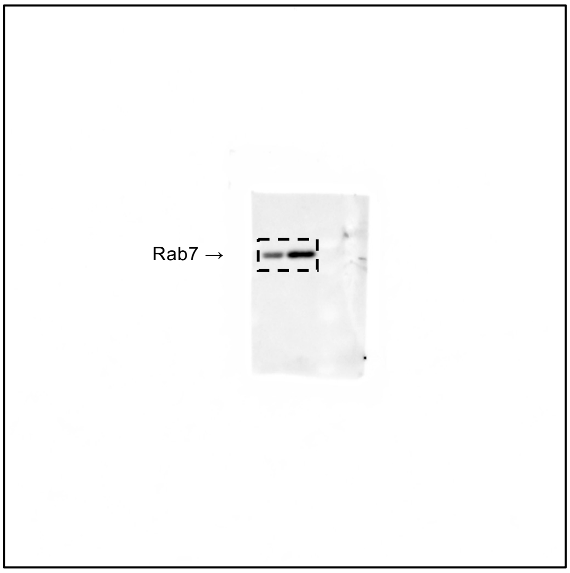

Full unedited image for Appendix Fig. S2b (left), Rab7.

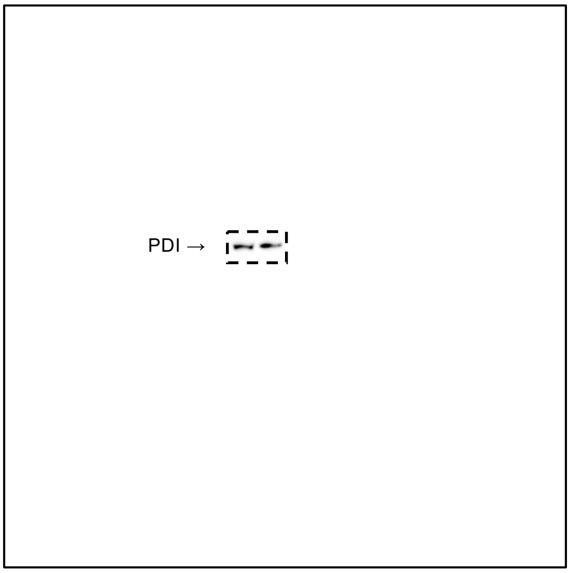

Full unedited image for Appendix Fig. S2b (left), PDI.

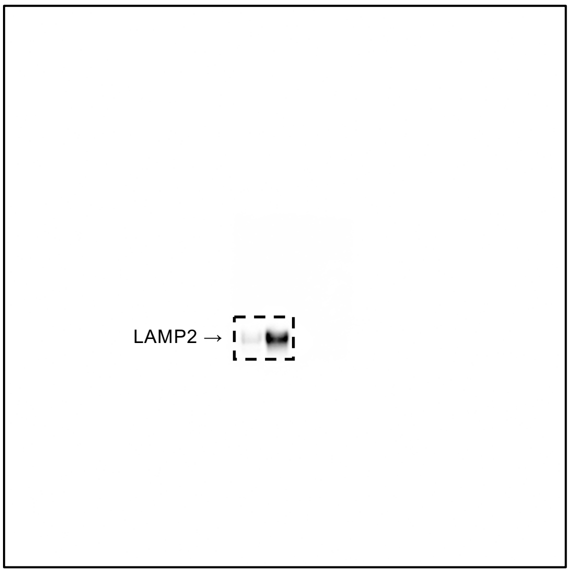

Full unedited image for Appendix Fig. S2b (left), LAMP2.

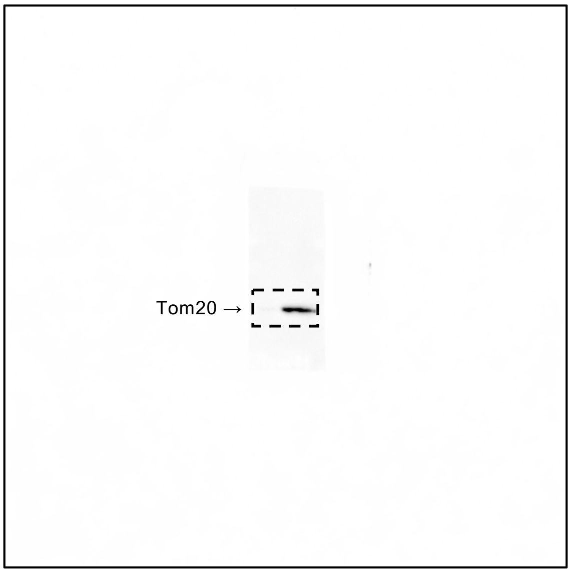

Full unedited image for Appendix Fig. S2b (left), Tom20.

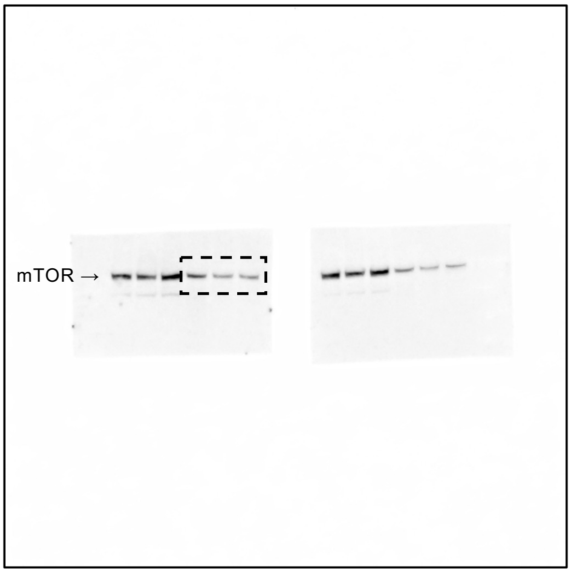

Full unedited image for Appendix Fig. S2b (right), mTOR.

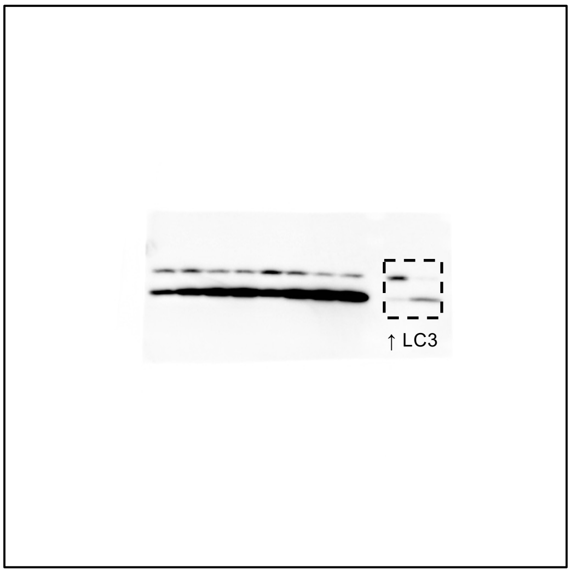

Full unedited image for Appendix Fig. S2b (left), LC3.

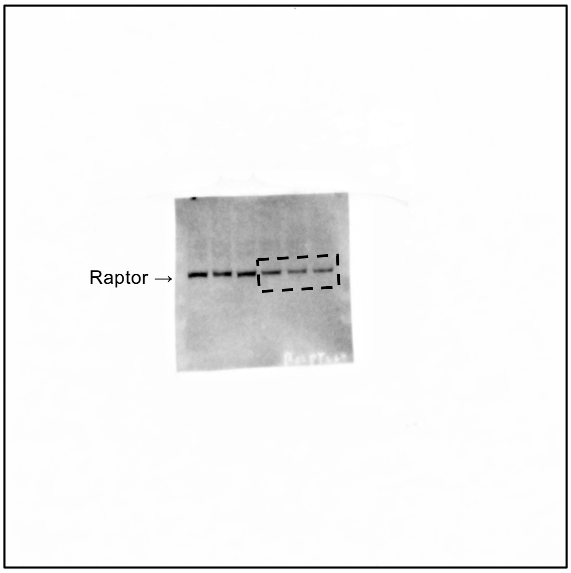

Full unedited image for Appendix Fig. S2b (right), Rapt[illegible]

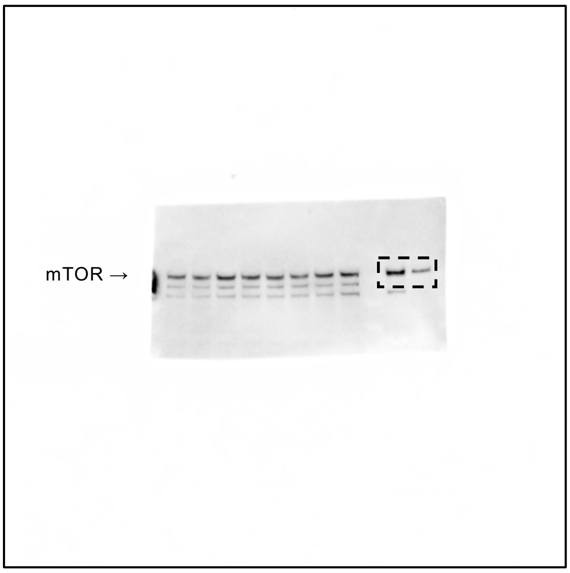

Full unedited image Appendix Fig. S2b (left), mTOR.

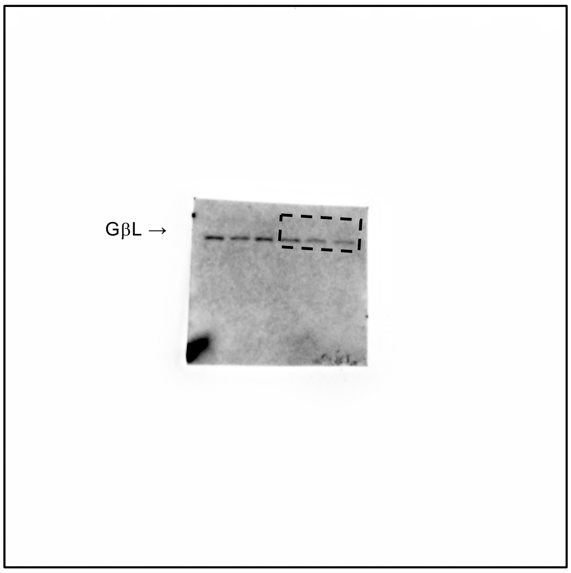

Full unedited image for Appendix Fig. S2b (right), GβL.

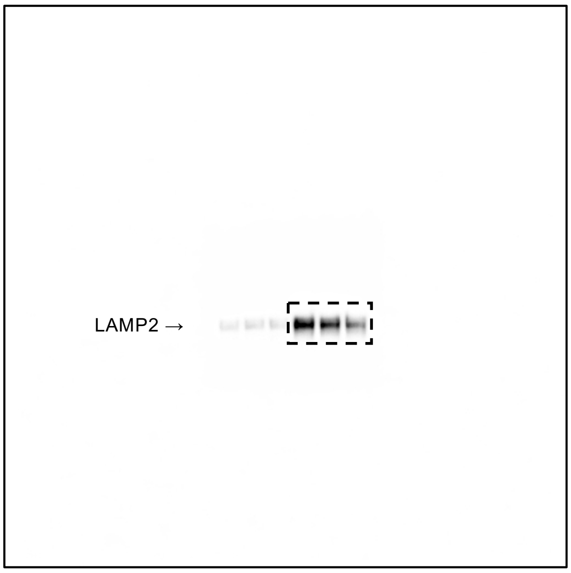

Full unedited image for Appendix Fig. S2b (right), LAMP2.

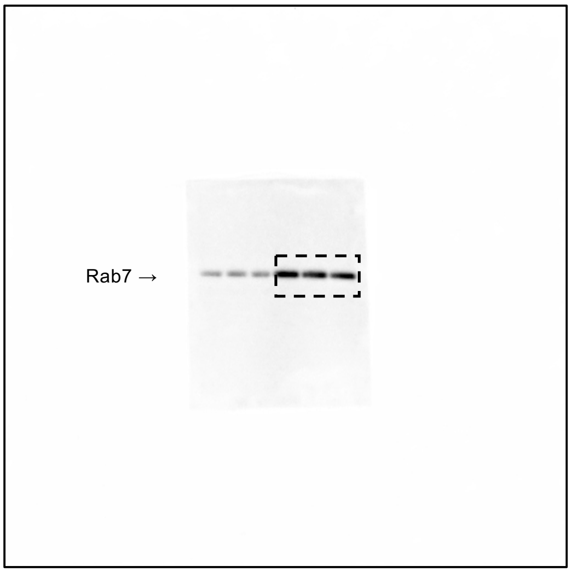

Full unedited image for Appendix Fig. S2b (right), Rab7.

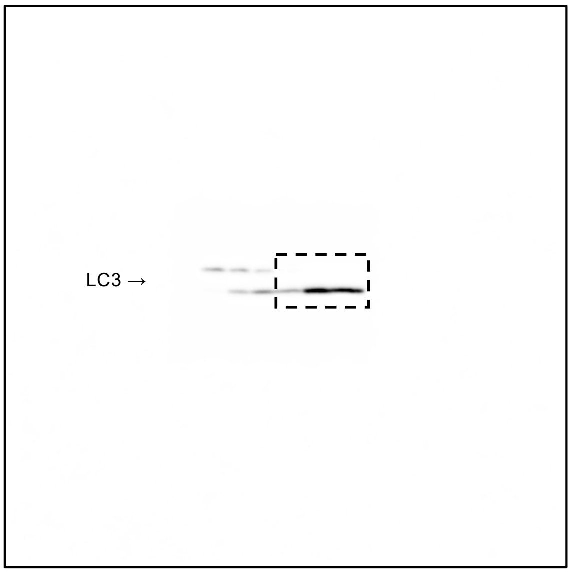

Full unedited image for Appendix Fig. S2b (right), LC3.

Source data for Appendix Fig. S3

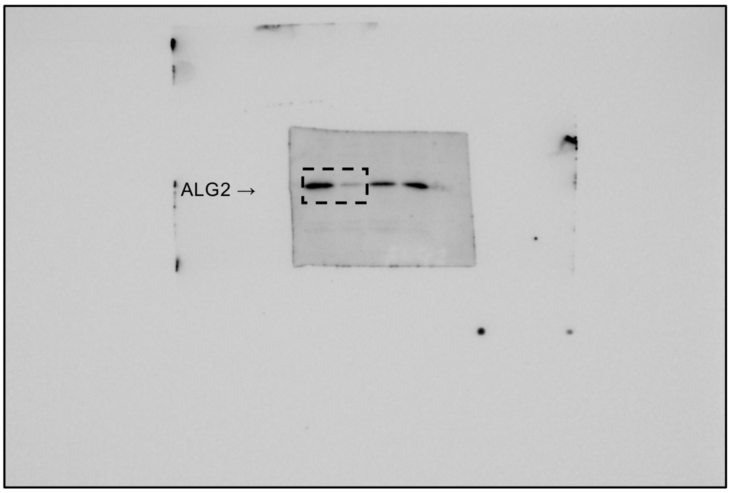

Full unedited image for Appendix Fig. S3c, ALG2.

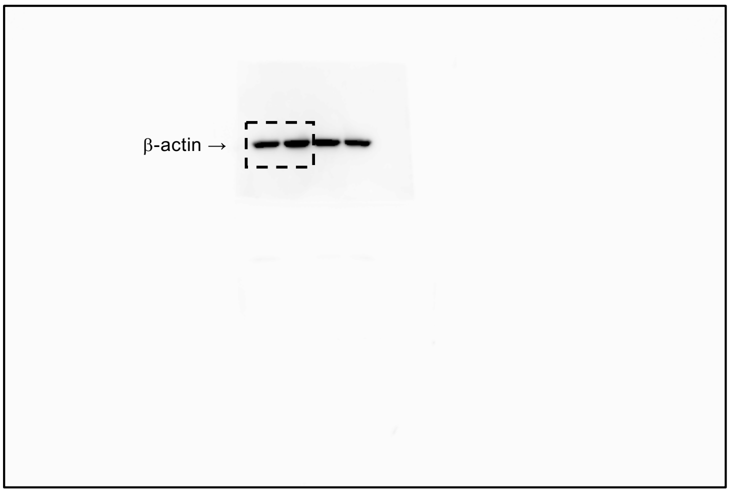

Full unedited image for Appendix Fig. S3c, β-actin.

Source data for Appendix Fig. S4

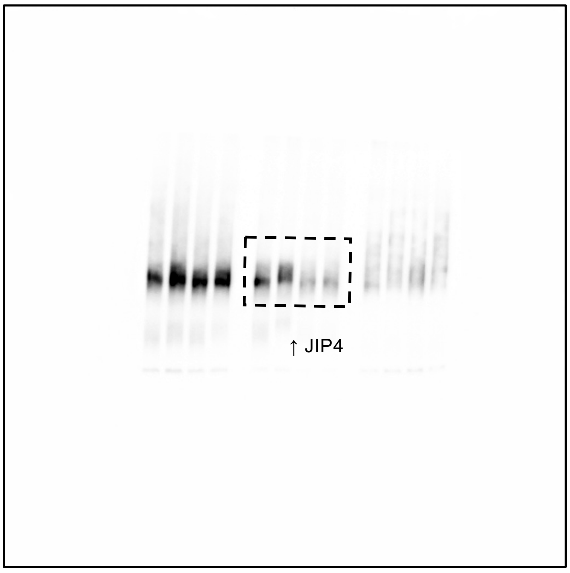

Full unedited image for Appendix Fig. S4b, JIP4.

Source data for Appendix Fig. S5

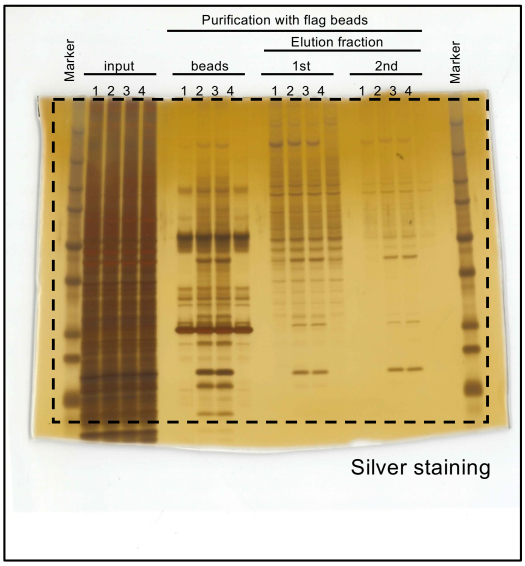

Full unedited image for Appendix Fig. S5a, Silver staining.

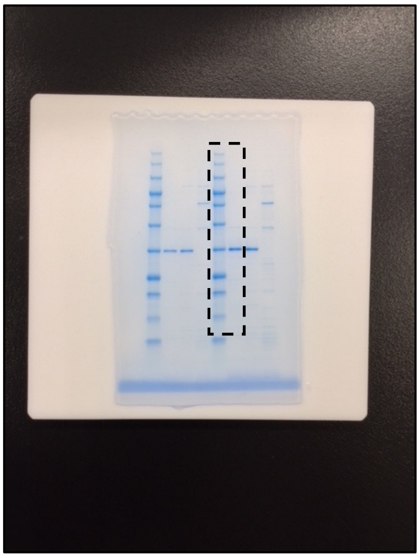

Full unedited image for Appendix Fig. S5b, CBB staining.

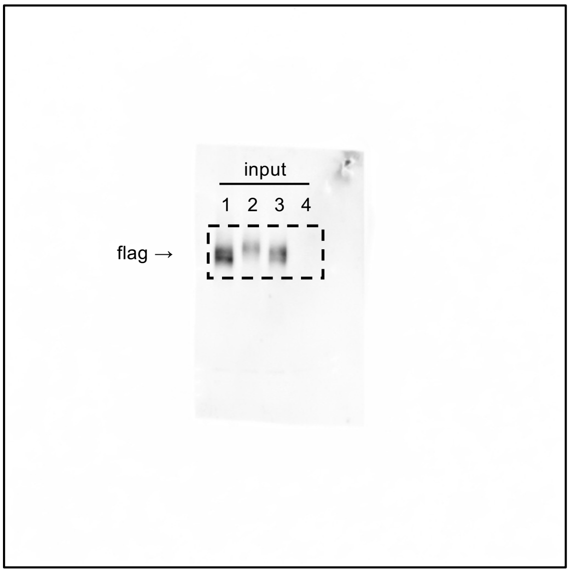

Full unedited image for Appendix Fig. S5a, flag.

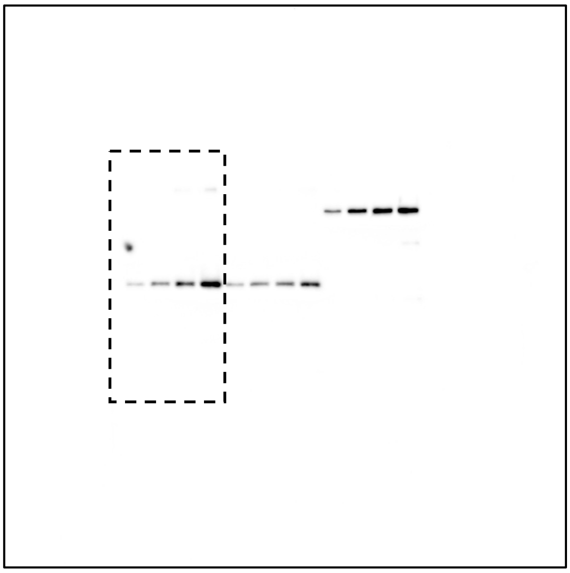

Full unedited image for Appendix Fig. S5b, His.

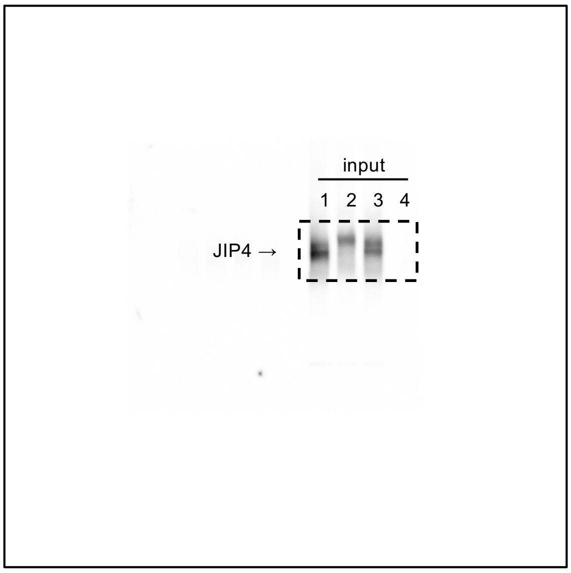

Full unedited image for Appendix Fig. S5a, JIP4.

Source data for Appendix Fig. S6

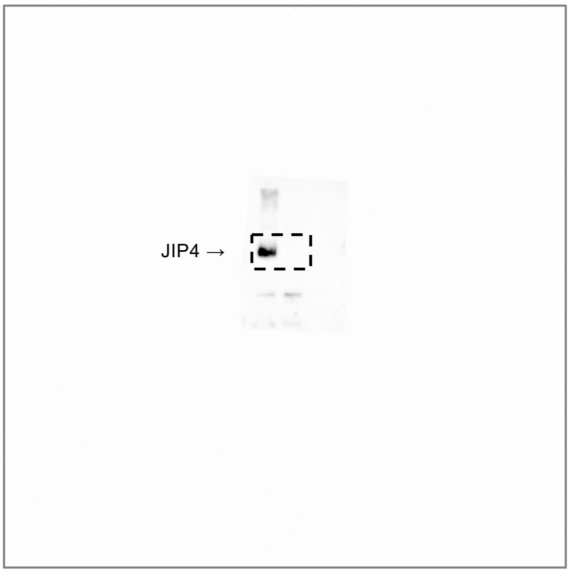

Full unedited image for Appendix Fig. S6b, JIP4.

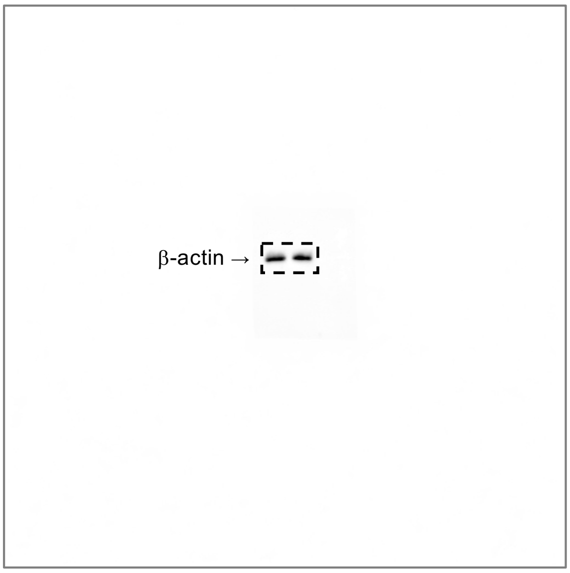

Full unedited image for Appendix Fig. S6b, β-actin.
