# Supplementary material for: Oxidative stress‐induced phosphorylation of JIP4 regulates lysosomal positioning in coordination with TRPML1 and ALG2
Source: EMBO J. 2022 Oct 11;41(22):e111476. doi: 10.15252/embj.2022111476 (PMC9670204; doi:10.15252/embj.2022111476)
Supplement: Supplementary file 6 — Source Data for Expanded View and Appendix [file EMBJ-41-e111476-s014.zip › Figure EV1/gel image_FigEV1.pdf]

# Source data for figure EV1

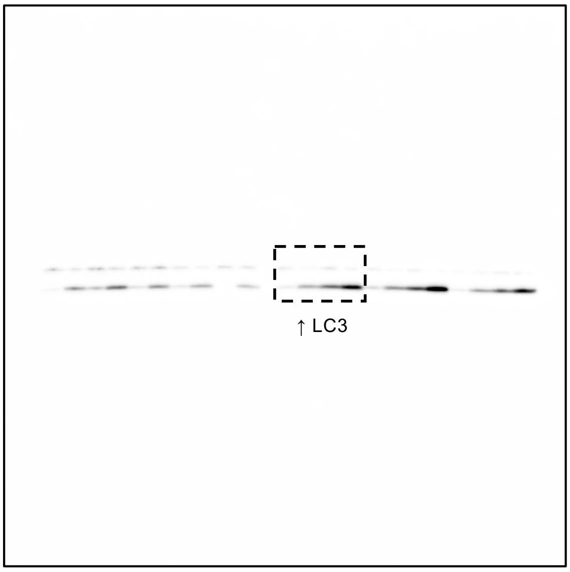


Full unedited image for Figure EV1a, LC3.

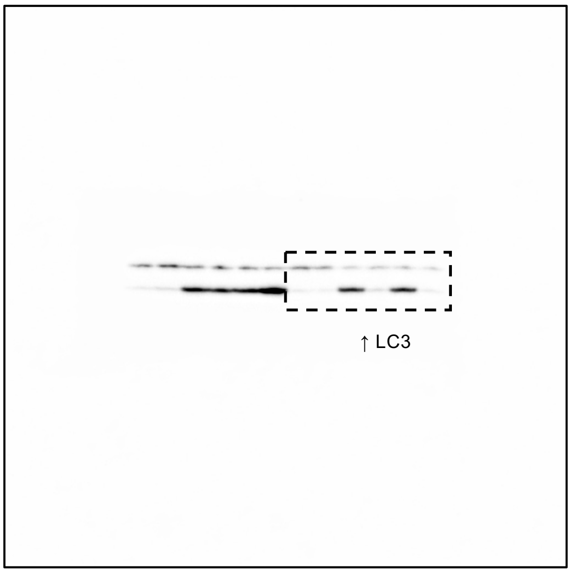


Full unedited image for Figure EV1b, LC3.

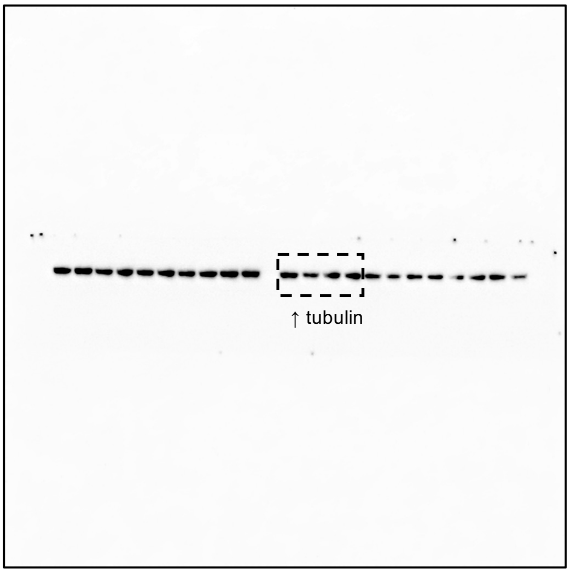


Full unedited image for Figure EV1a, tubulin.

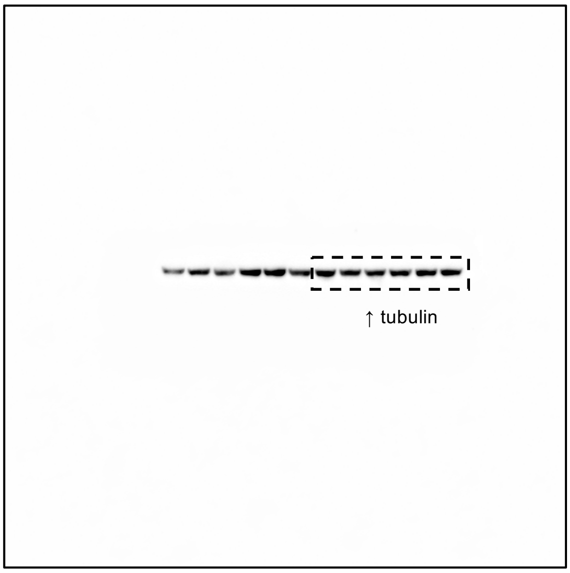


Full unedited image for Figure EV1b, tubulin.
