# Supplementary material for: Oxidative stress‐induced phosphorylation of JIP4 regulates lysosomal positioning in coordination with TRPML1 and ALG2
Source: EMBO J. 2022 Oct 11;41(22):e111476. doi: 10.15252/embj.2022111476 (PMC9670204; doi:10.15252/embj.2022111476)
Supplement: Supplementary file 6 — Source Data for Expanded View and Appendix [file EMBJ-41-e111476-s014.zip › Figure EV2/gel image_FigEV2.pdf]

Source data for figure EV2

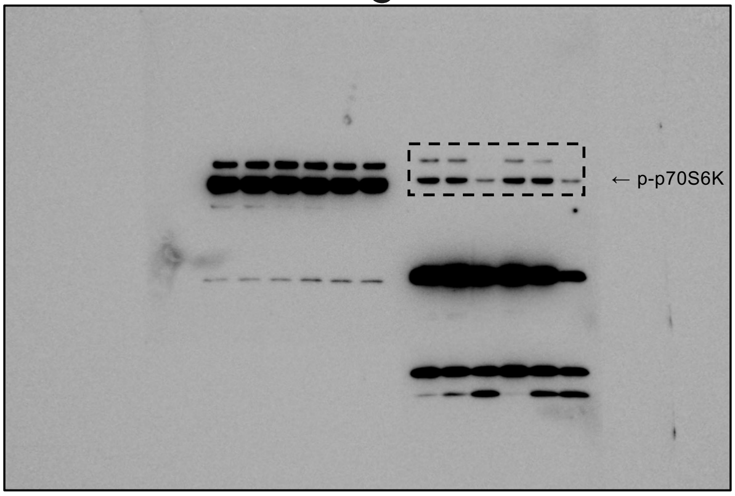

Full unedited image for Figure EV2a, phospho-p70S6K.

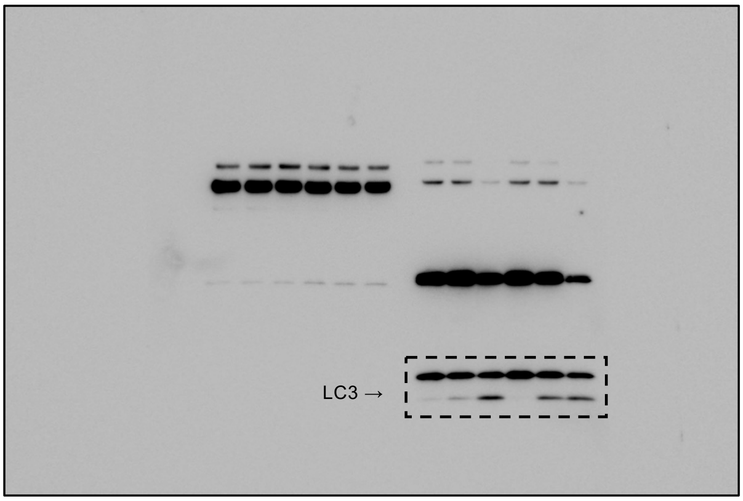

Full unedited image for Figure EV2a, LC3.

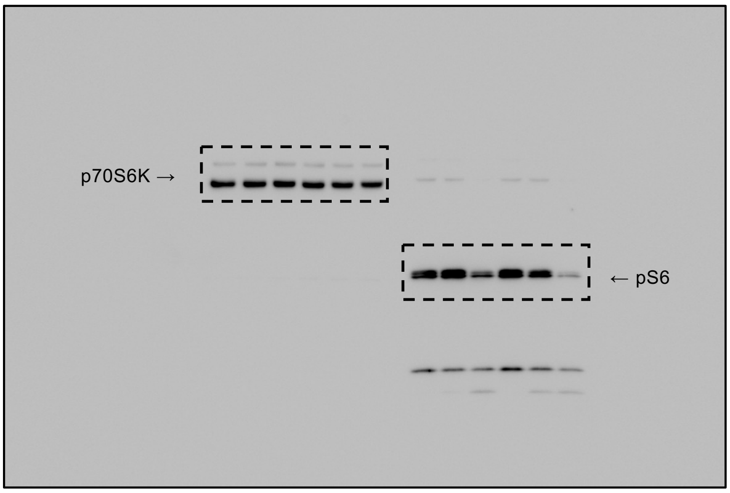

Full unedited image for Figure EV2a, p70S6K and phospho-S6.

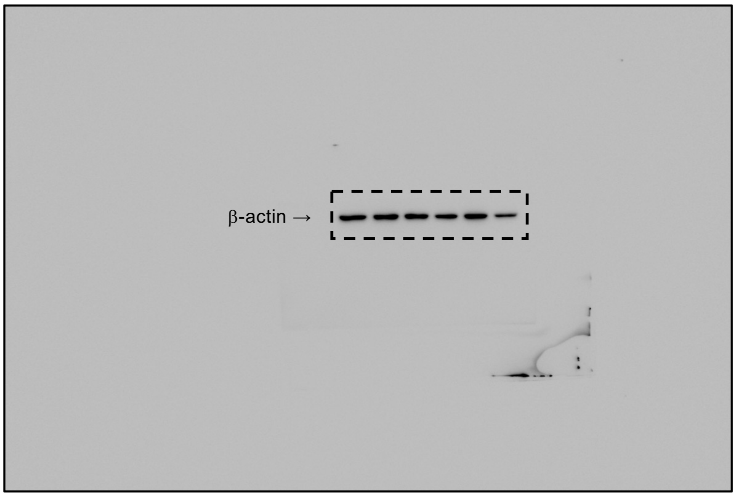

Full unedited image for Figure EV2a, β-actin.

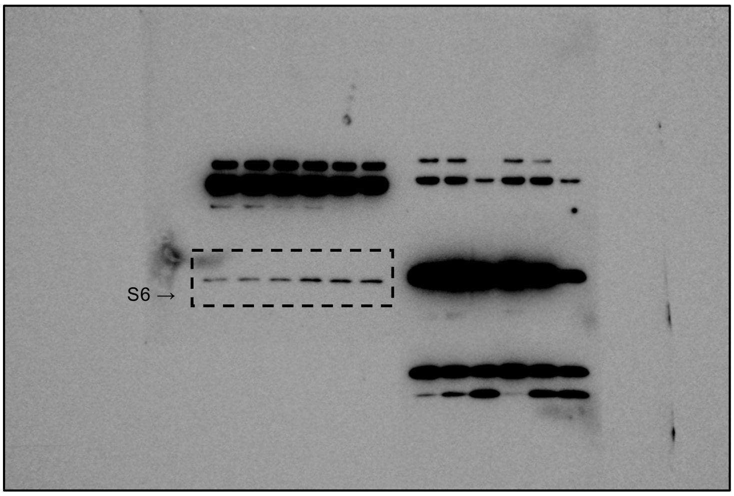

Full unedited image for Figure EV2a, S6.

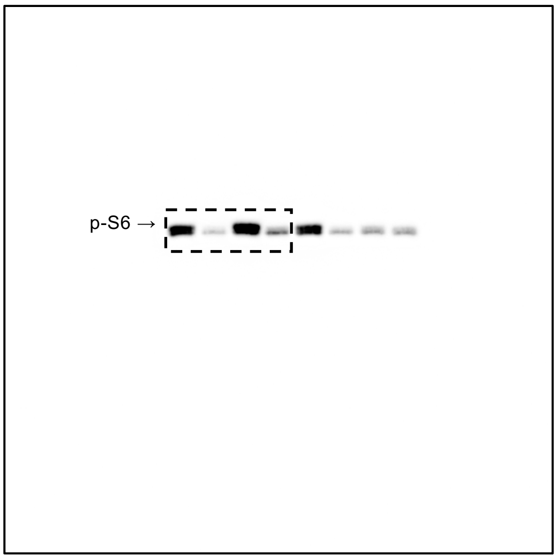

Full unedited image for Figure EV2b, p-S6

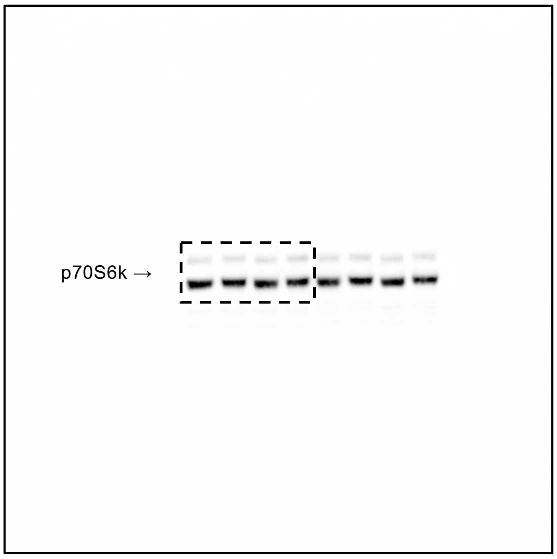

Full unedited image for Figure EV2b, p70S6k

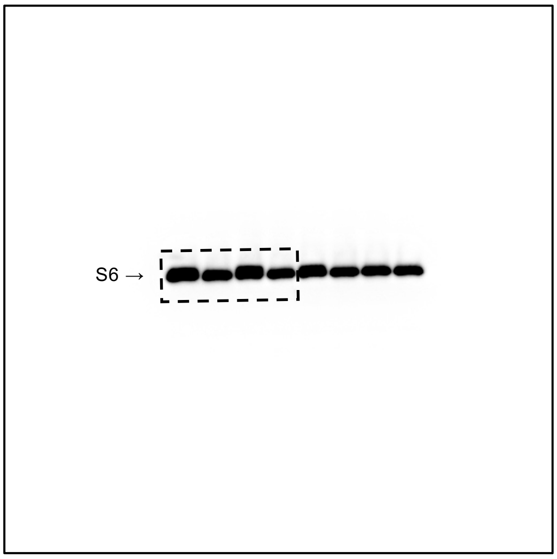

Full unedited image for Figure EV2b, S6

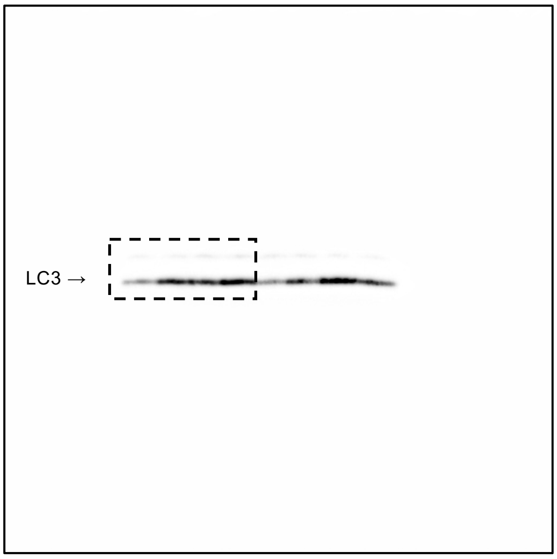

Full unedited image for Figure EV2b, LC3

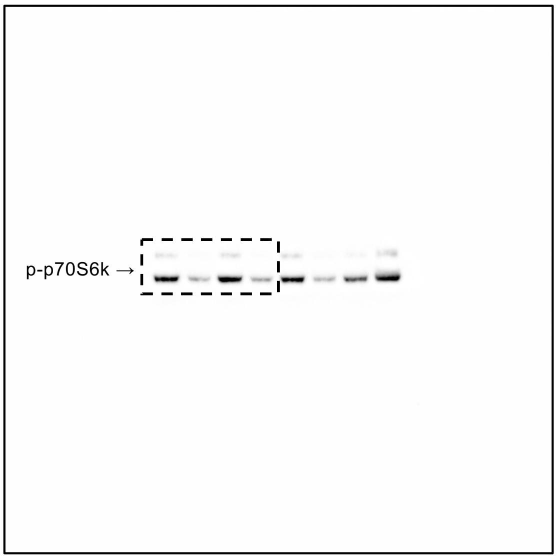

Full unedited image for Figure EV2b, p-p70S6k

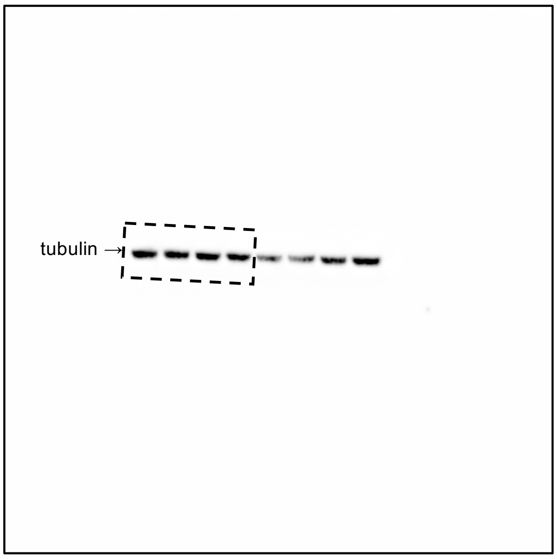

Full unedited image for Figure EV2b, tubulin

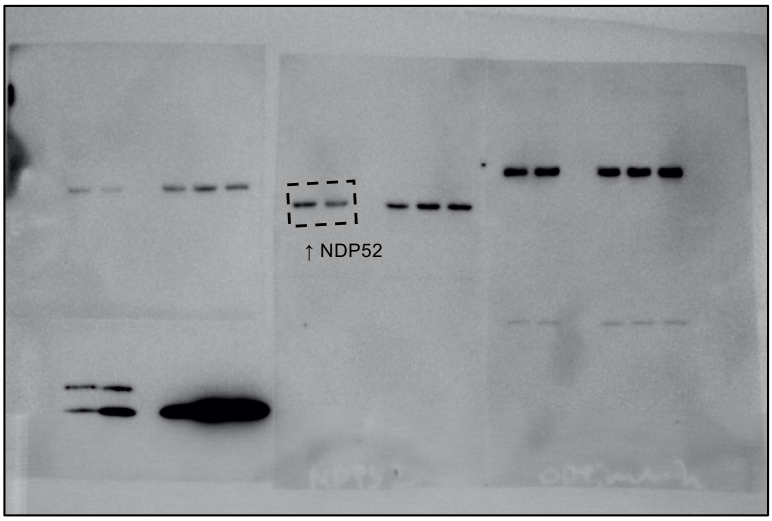

Full unedited image for Figure EV2e, NDP52.

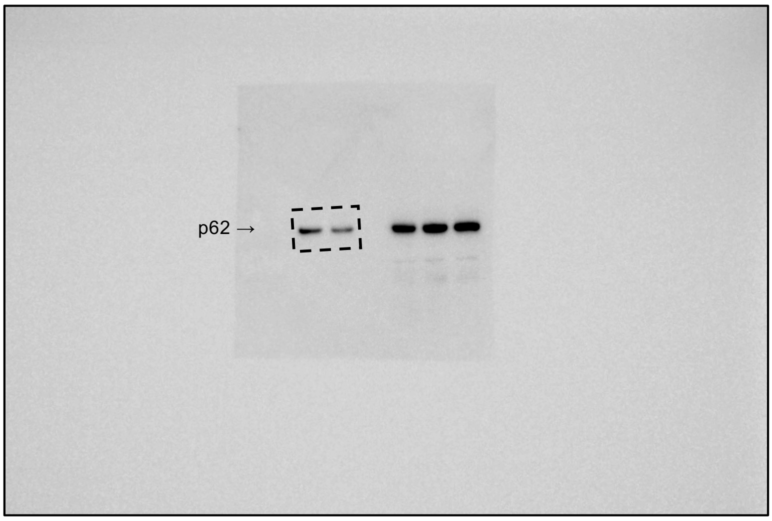

Full unedited image for Figure EV2e, p62.

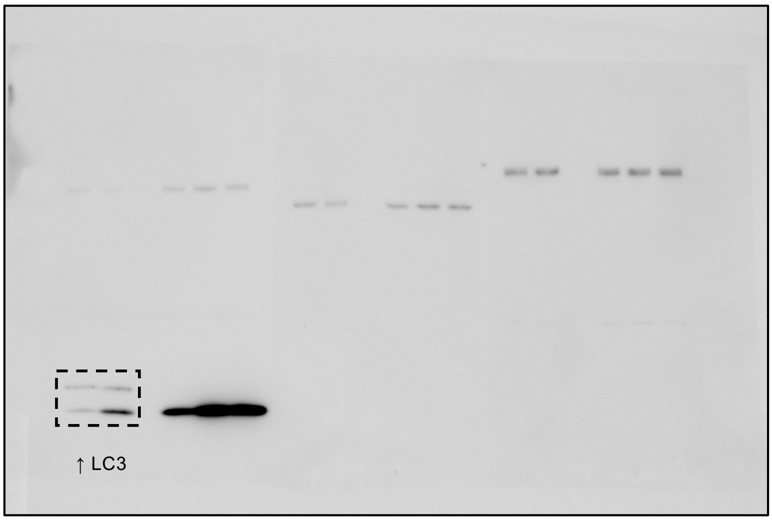

Full unedited image for Figure EV2e, LC3.
