# Supplementary material for: Oxidative stress‐induced phosphorylation of JIP4 regulates lysosomal positioning in coordination with TRPML1 and ALG2
Source: EMBO J. 2022 Oct 11;41(22):e111476. doi: 10.15252/embj.2022111476 (PMC9670204; doi:10.15252/embj.2022111476)
Supplement: Supplementary file 6 — Source Data for Expanded View and Appendix [file EMBJ-41-e111476-s014.zip › Figure EV3/gel image_FigEV3.pdf]

Source data for figure EV3

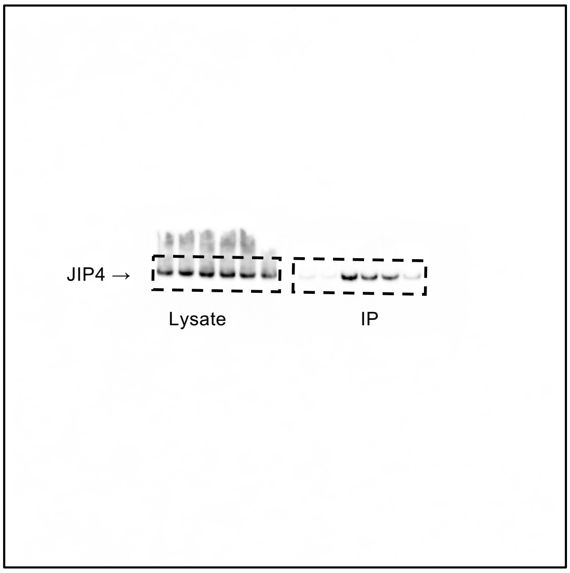

Full unedited image for Figure EV3a, JIP4 (Lysate/IP).

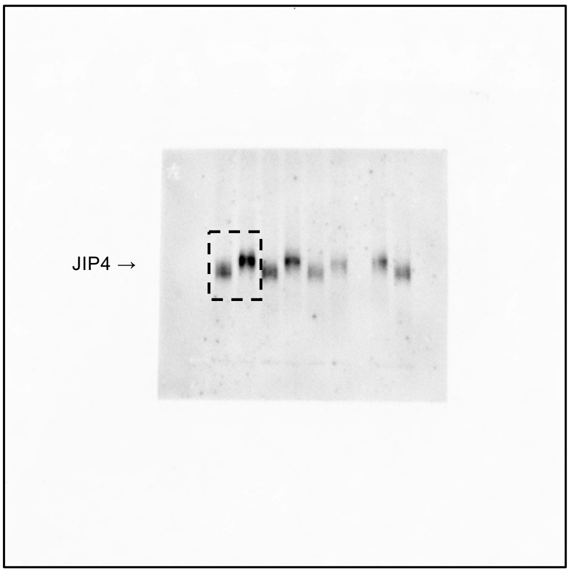

Full unedited image for Figure EV3d, JIP4.

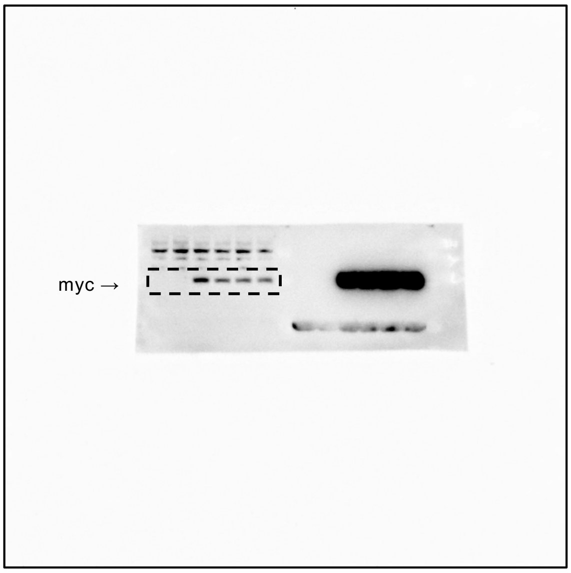

Full unedited image for Figure EV3a, myc (Lysate).

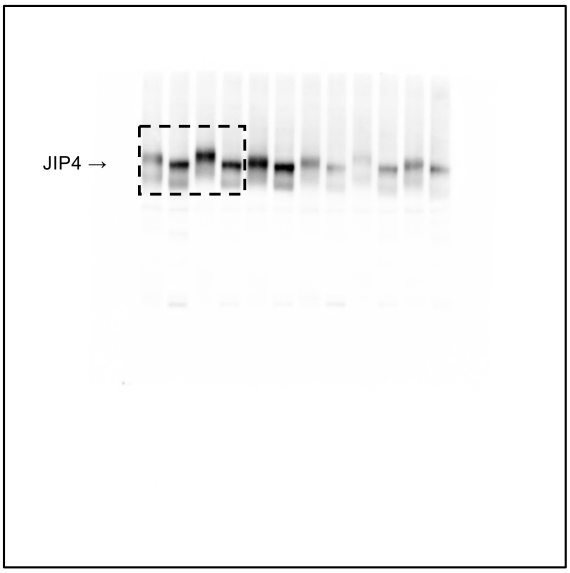

Full unedited image for Figure EV3e, JIP4.

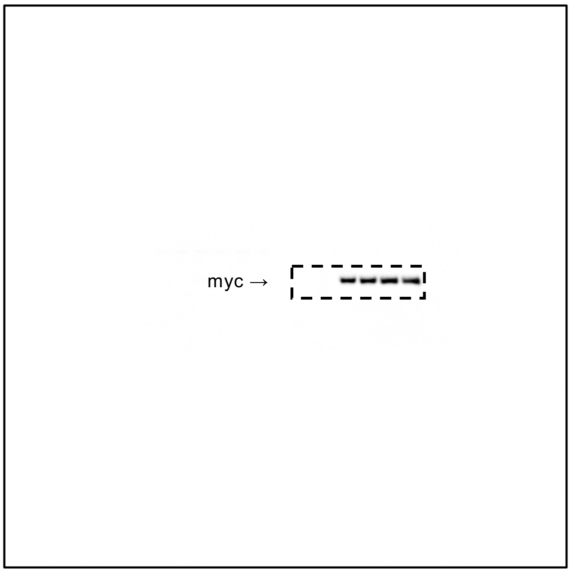

Full unedited image for Figure EV3a, myc (IP).

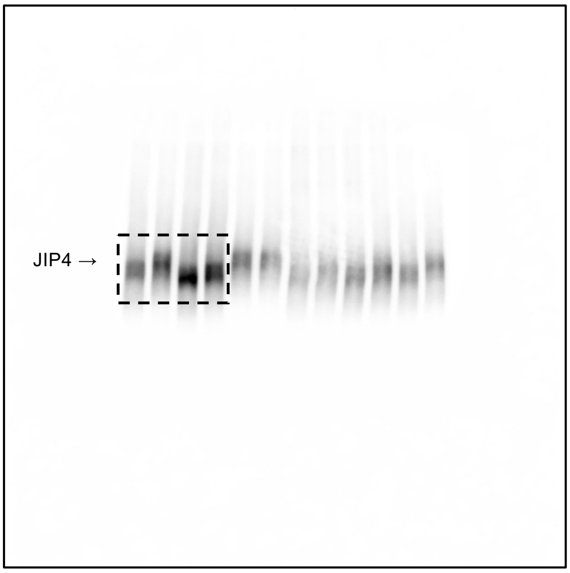

Full unedited image for Figure EV3h, JIP4.

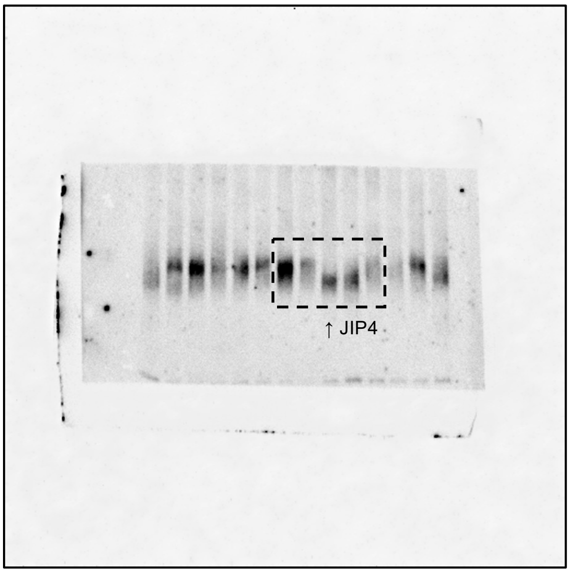

Full unedited image for Figure EV3k, JIP4.
