# Supplementary material for: Oxidative stress‐induced phosphorylation of JIP4 regulates lysosomal positioning in coordination with TRPML1 and ALG2
Source: EMBO J. 2022 Oct 11;41(22):e111476. doi: 10.15252/embj.2022111476 (PMC9670204; doi:10.15252/embj.2022111476)
Supplement: Supplementary file 6 — Source Data for Expanded View and Appendix [file EMBJ-41-e111476-s014.zip › Figure EV4/gel image_FigEV4.pdf]

Source data for figure EV4

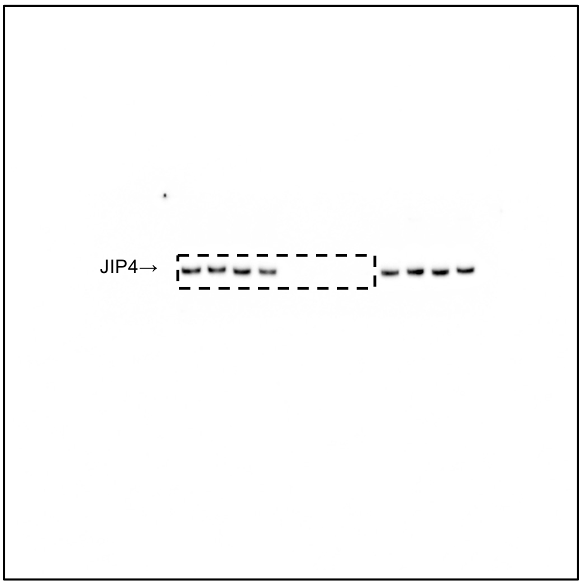

Full unedited image for Figure EV4a, JIP4 (short exposure).

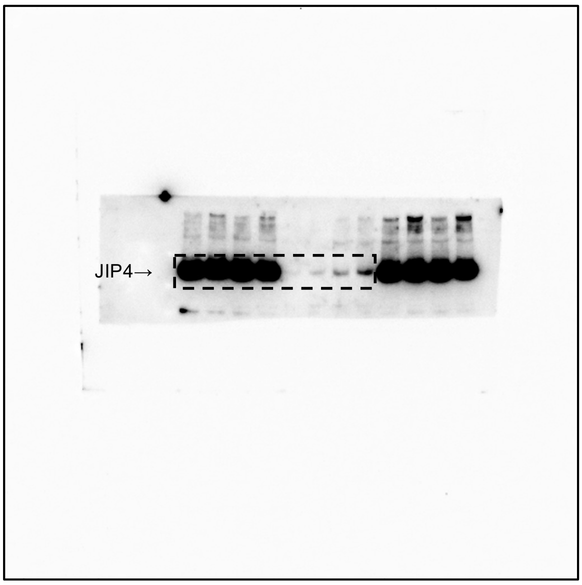

Full unedited image for Figure EV4a, JIP4 (long exposure).

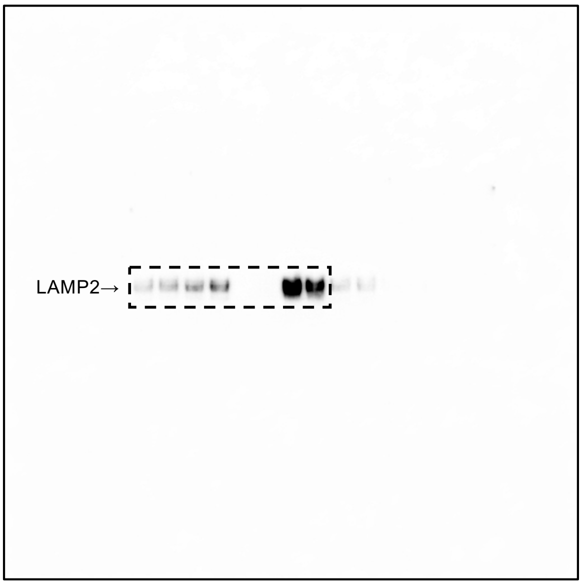

Full unedited image for Figure EV4a, LAMP2.

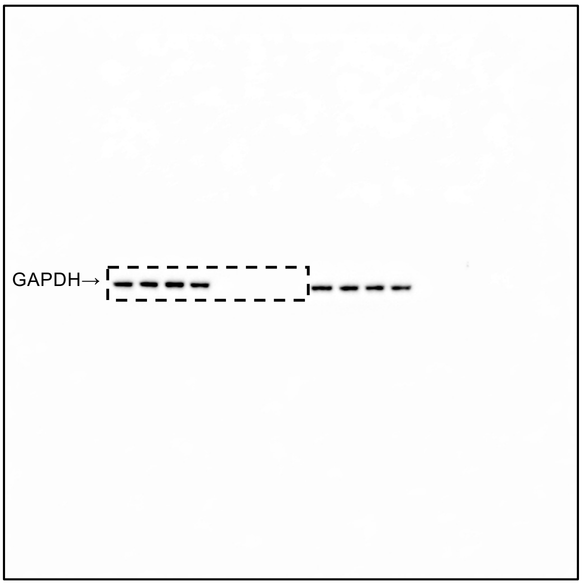

Full unedited image for Figure EV4a, GAPDH
