# Supplementary material for: Oxidative stress‐induced phosphorylation of JIP4 regulates lysosomal positioning in coordination with TRPML1 and ALG2
Source: EMBO J. 2022 Oct 11;41(22):e111476. doi: 10.15252/embj.2022111476 (PMC9670204; doi:10.15252/embj.2022111476)
Supplement: Supplementary file 8 — Source Data for Figure 2 [file EMBJ-41-e111476-s008.zip › gel image_Fig2.pdf]

Source data for figure 2

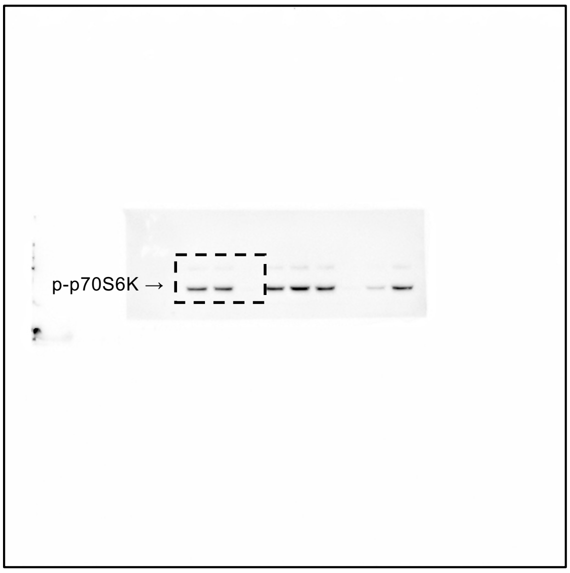

Full unedited image for Figure 2e, p-p70S6K.

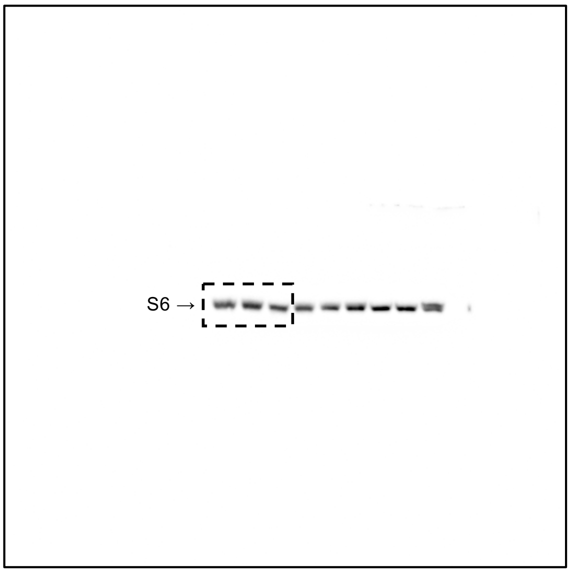

Full unedited image for Figure 2e, S6.

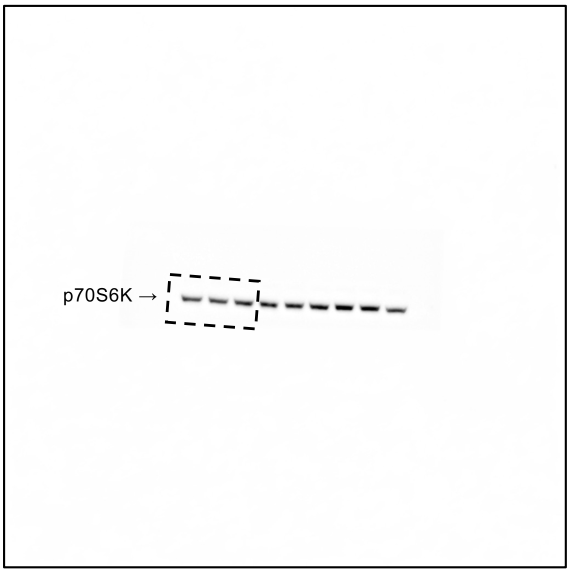

Full unedited image for Figure 2e, p70S6K.

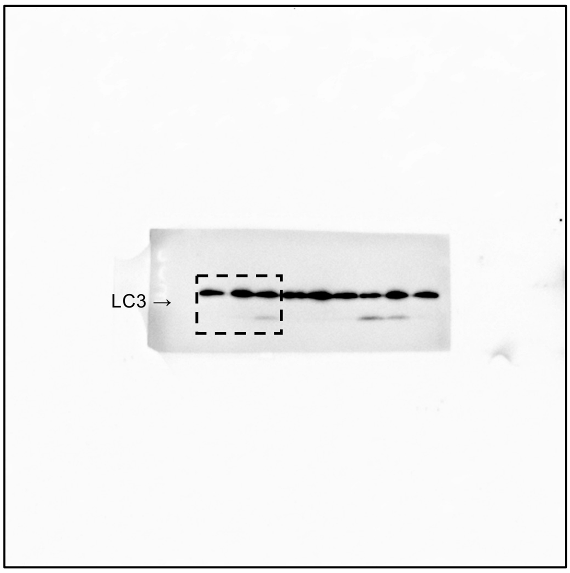

Full unedited image for Figure 2e, LC3.

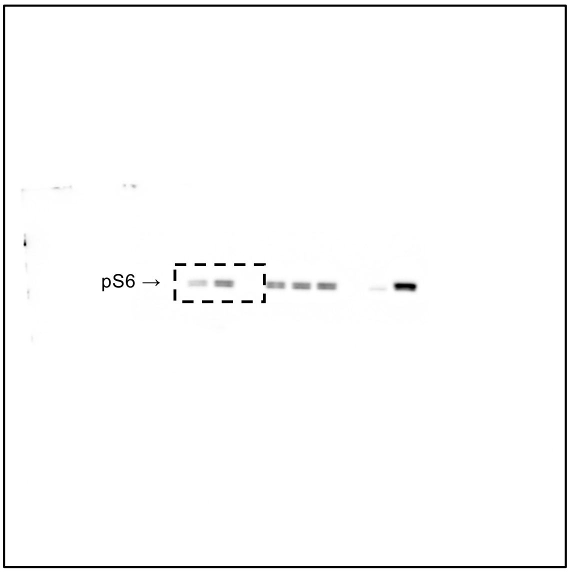

Full unedited image for Figure 2e, p-S6.

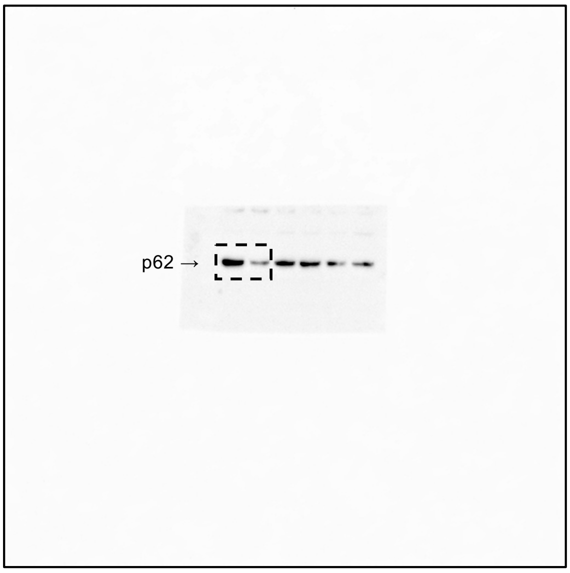

Full unedited image for Figure 2h, p62.

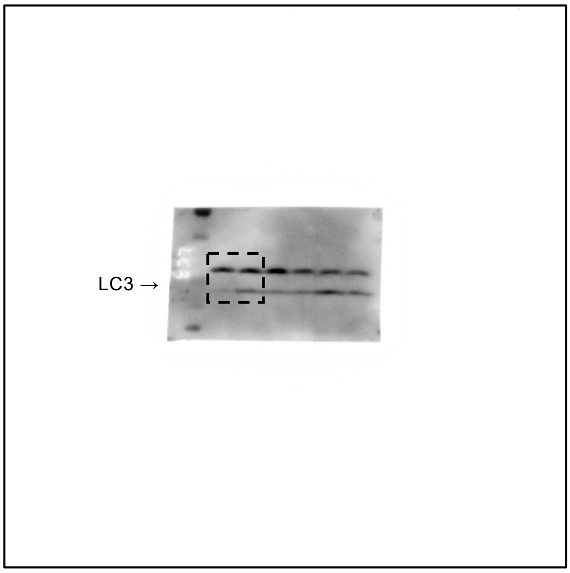

Full unedited image for Figure 2h, LC3.

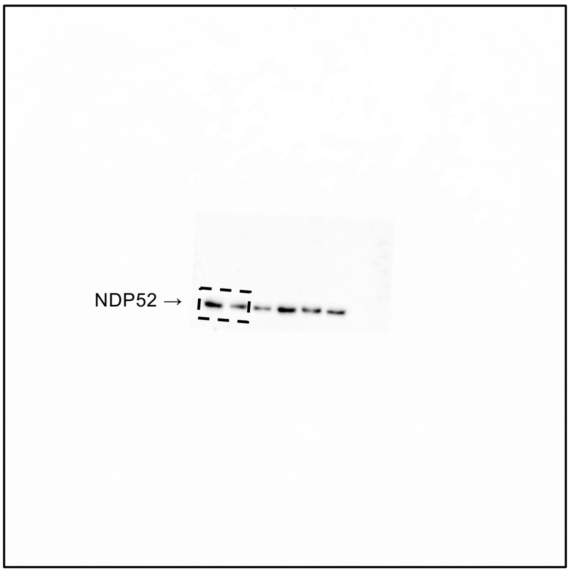

Full unedited image for Figure 2h, NDP52.

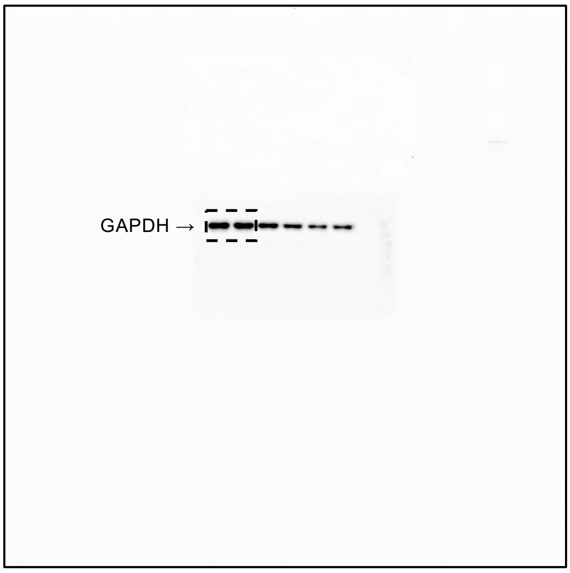

Full unedited image for Figure 2h, GAPDH.
