# Supplementary material for: Oxidative stress‐induced phosphorylation of JIP4 regulates lysosomal positioning in coordination with TRPML1 and ALG2
Source: EMBO J. 2022 Oct 11;41(22):e111476. doi: 10.15252/embj.2022111476 (PMC9670204; doi:10.15252/embj.2022111476)
Supplement: Supplementary file 10 — Source Data for Figure 4 [file EMBJ-41-e111476-s013.zip › gel image_Fig4.pdf]

Source data for figure 4

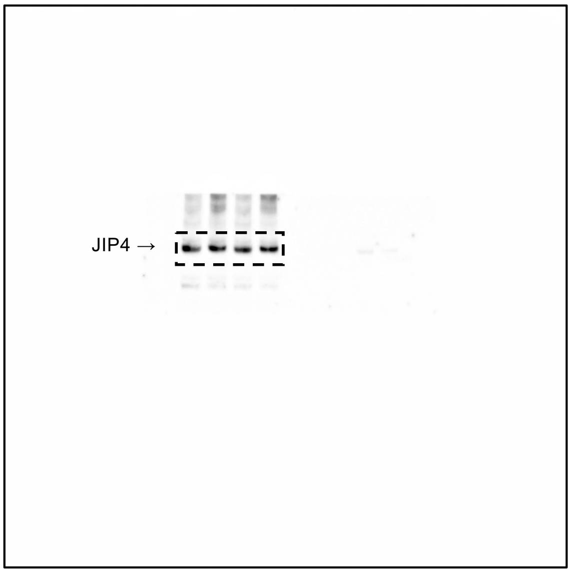

Full unedited image for Figure 4a, JIP4 (Lysate).

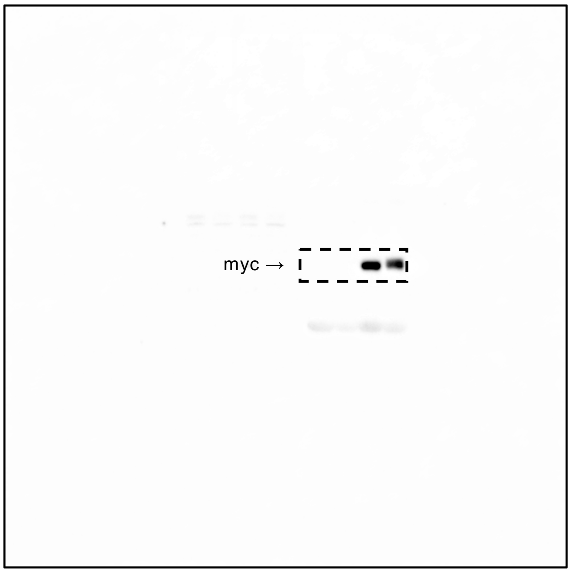

Full unedited image for Figure 4a, myc (IP).

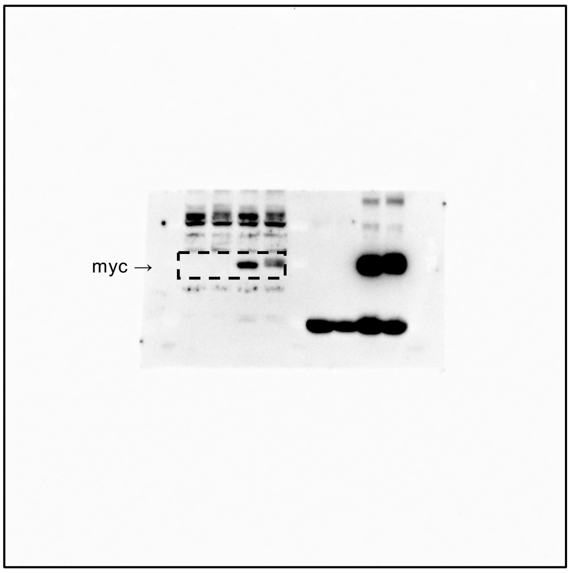

Full unedited image for Figure 4a, myc (Lysate).

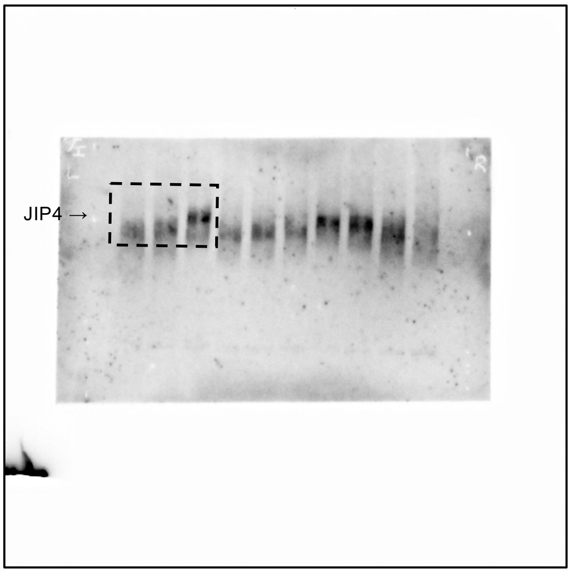

Full unedited image for Figure 4c, JIP4.

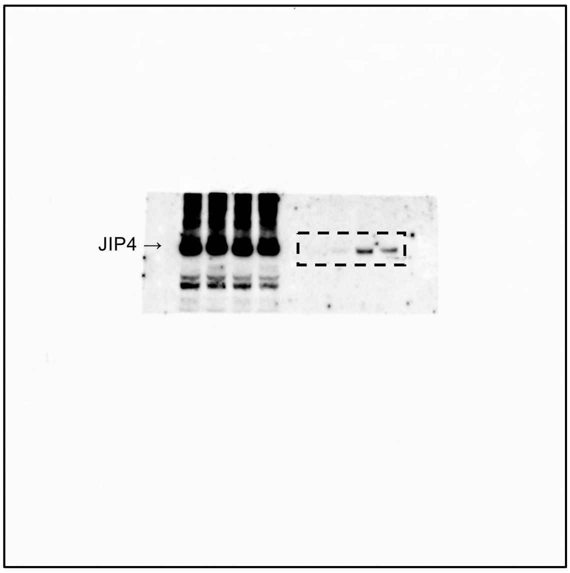

Full unedited image for Figure 4a, JIP4 (IP).

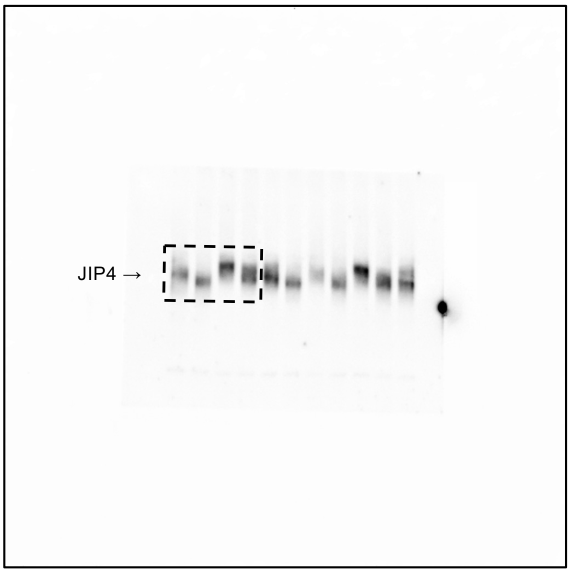

Full unedited image for Figure 4d, JIP4.

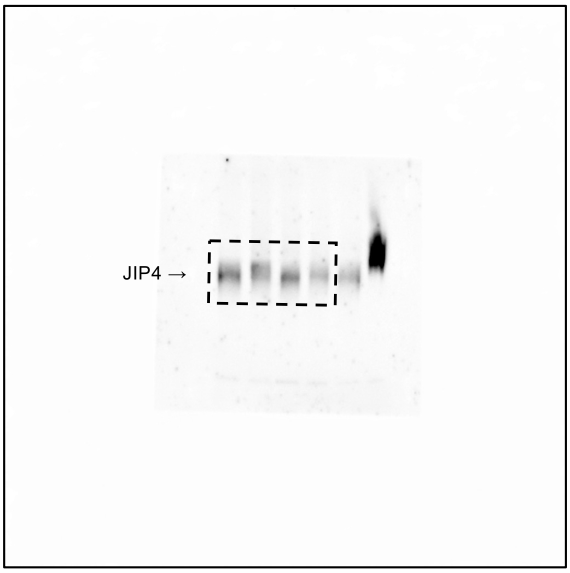

Full unedited image for Figure 4f, JIP4.

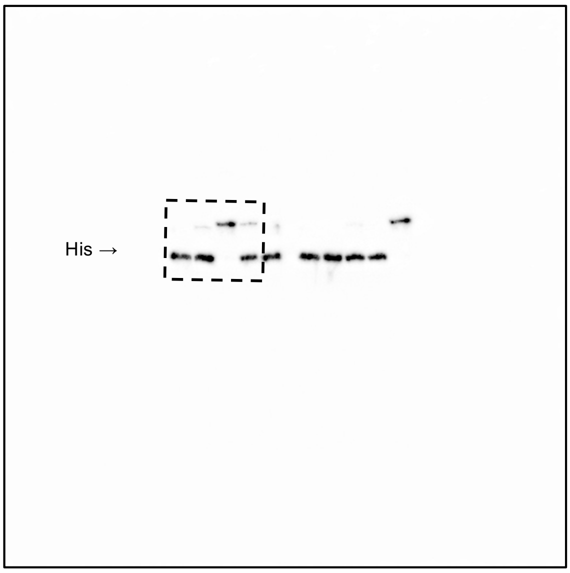

Full unedited image for Figure 4j, His
