# Supplementary material for: Oxidative stress‐induced phosphorylation of JIP4 regulates lysosomal positioning in coordination with TRPML1 and ALG2
Source: EMBO J. 2022 Oct 11;41(22):e111476. doi: 10.15252/embj.2022111476 (PMC9670204; doi:10.15252/embj.2022111476)
Supplement: Supplementary file 11 — Source Data for Figure 5 [file EMBJ-41-e111476-s005.zip › gel image_Fig5.pdf]

Source data for figure 5

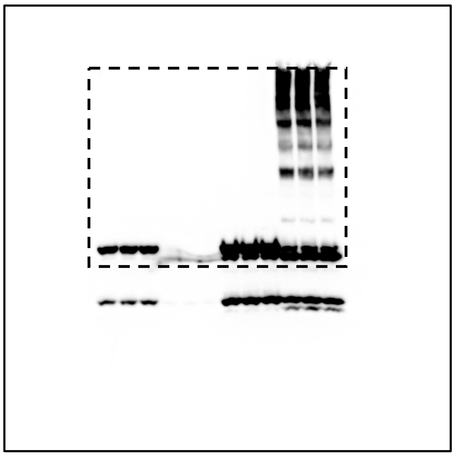

Full unedited image for Figure 5c, RFP

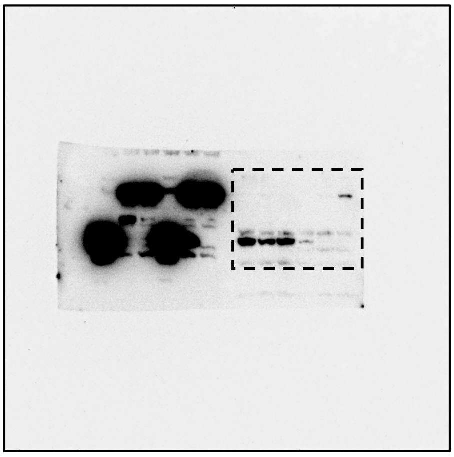

Full unedited image for Figure 5c, GFP (IP)

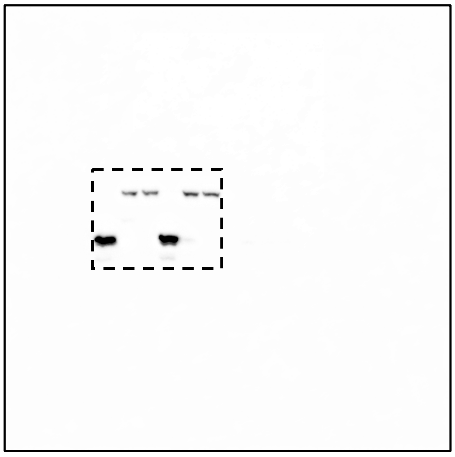

Full unedited image for Figure 5c, GFP (input)
