# Supplementary material for: Oxidative stress‐induced phosphorylation of JIP4 regulates lysosomal positioning in coordination with TRPML1 and ALG2
Source: EMBO J. 2022 Oct 11;41(22):e111476. doi: 10.15252/embj.2022111476 (PMC9670204; doi:10.15252/embj.2022111476)
Supplement: Supplementary file 12 — Source Data for Figure 6 [file EMBJ-41-e111476-s010.zip › gel image_Fig6.pdf]

Source data for figure 6

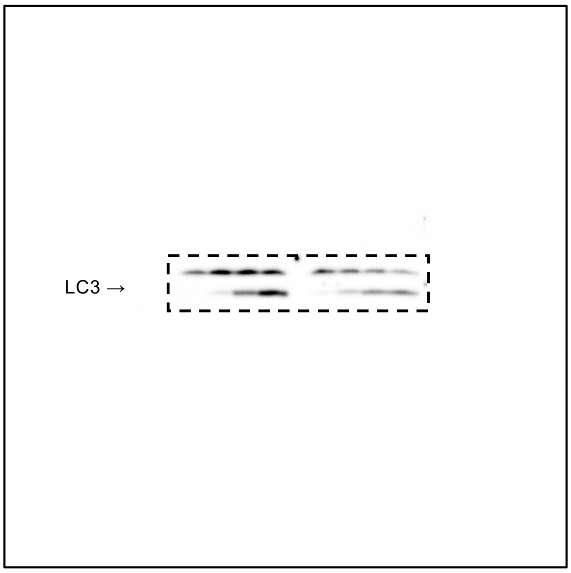

Full unedited image for Figure 6a, LC3.

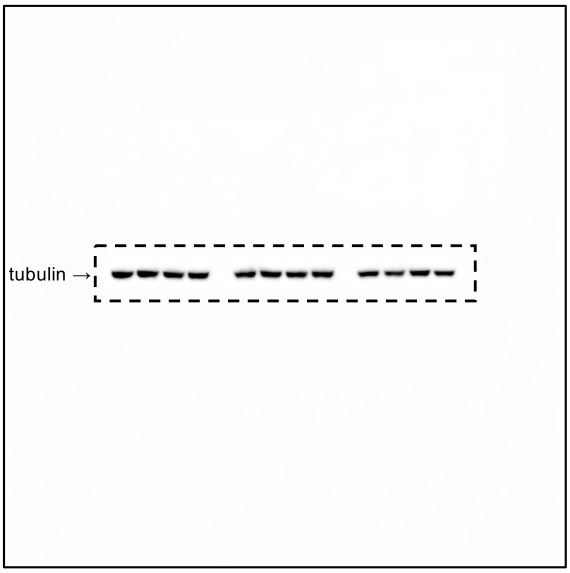

Full unedited image for Figure 6b, tubulin.

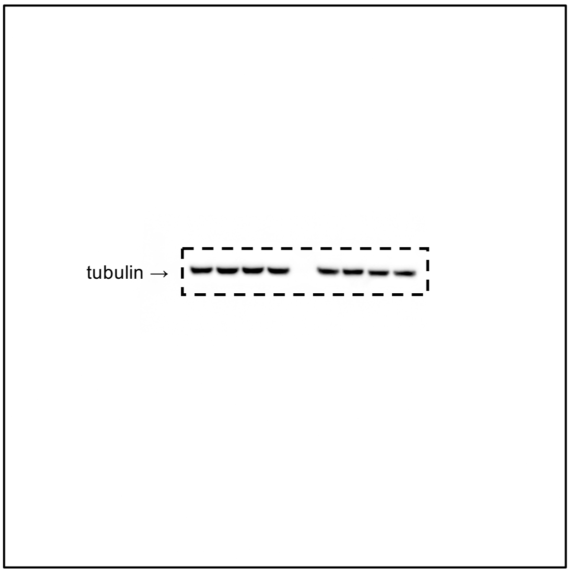

Full unedited image for Figure 6a tubulin.

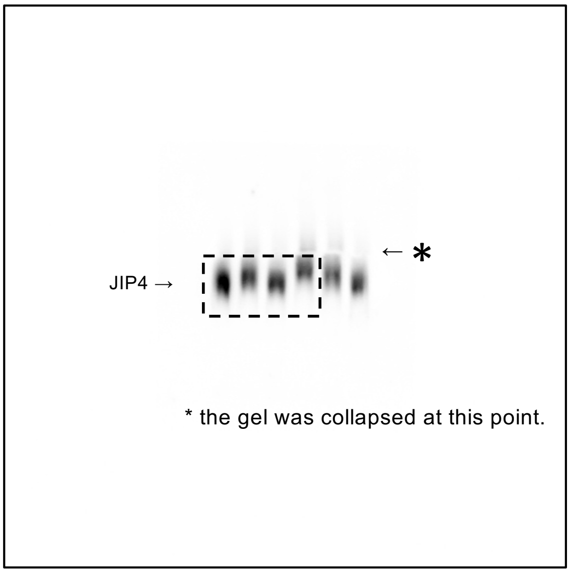

Full unedited image for Figure 6e, JIP4 (left).

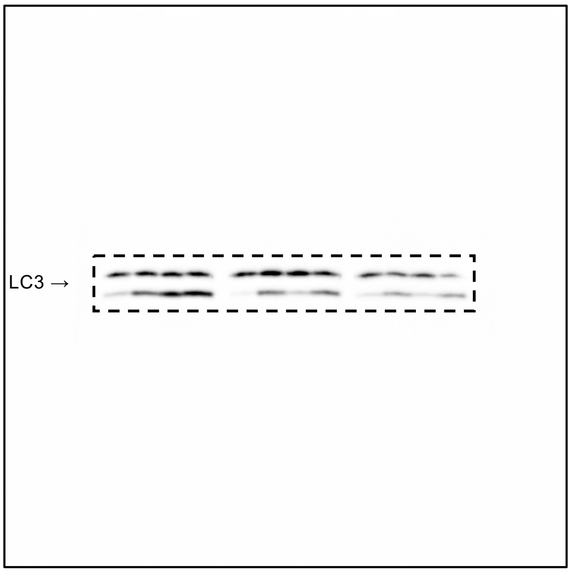

Full unedited image for Figure 6b, LC3.

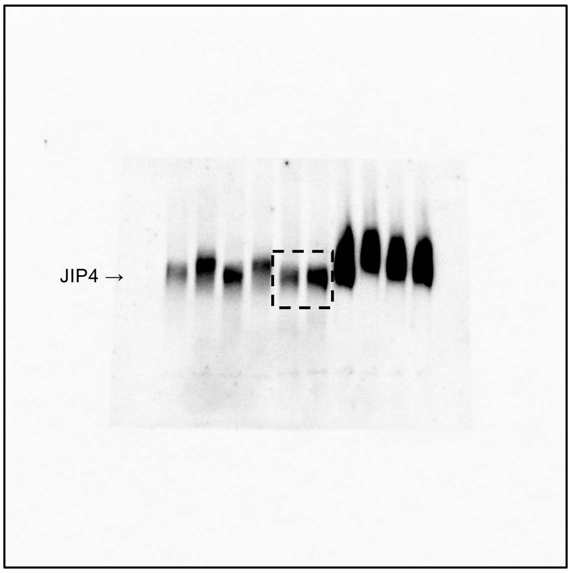

Full unedited image for Figure 6e, JIP4 (right).
